# Identification and validation of parthanatos-related genes in lung adenocarcinoma and construction of a prognostic risk model

**DOI:** 10.3389/fimmu.2026.1806560

**Published:** 2026-07-08

**Authors:** Yanna Yang, Jie Liu, Bin Shang, Shujuan Jiang

**Affiliations:** 1 Department of Pulmonary and Critical Care Medicine, Jinan Central Hospital, Shandong University, Jinan, China; 2 Department of Pulmonary and Critical Care Medicine, Central Hospital Affiliated to Shandong First Medical University, Jinan, China; 3 Department of Oncology, Central Hospital Affiliated to Shandong First Medical University, Jinan, China; 4 Department of Thoracic Surgery, Shandong Provincial Hospital Affiliated to Shandong First Medical University, Jinan, China; 5 Department of Pulmonary and Critical Care Medicine, Shandong Provincial Hospital, Shandong University, Jinan, China

**Keywords:** lung adenocarcinoma, machine learning, parthanatos, prognostic risk model, single-cell RNA sequencing

## Abstract

**Background:**

As a major cause of cancer-related death, lung adenocarcinoma (LUAD) remains a significant health challenge. Parthanatos plays a crucial role in tumor progression, influencing cancer cell survival and therapy resistance. This study constructed a parthanatos-based prognostic model and explored its associated biological processes.

**Methods:**

Data on LUAD transcriptomics and parthanatos-related genes were collected from publicly databases and related literature. Differential expression analysis and weighted gene co-expression network analysis were employed to identify candidate genes. Univariate Cox regression analysis and machine learning algorithms were employed to screen prognostic genes and construct the risk model. Gene expression patterns and intercellular communication within distinct cell types were explored by single-cell sequencing. Lastly, prognostic gene expression in tissue samples was verified by reverse transcription quantitative polymerase chain reaction (RT-qPCR) and western blotting.

**Results:**

*PPP1R14B*, *MIF*, *ALG3*, *C11orf24*, and *MZT2A* were identified as prognostic genes. The risk model had good predictive performance. The risk score effectively stratified patients into high- and low-risk groups with significantly divergent overall survival (p< 0.0001). Prognostic genes were involved in vital processes such as DNA replication, protein metabolism, and immune response. Single-cell analysis highlighted expression variations across cell types, particularly in epithelial cells, with strong communication from myeloid cells and fibroblasts. RT-qPCR confirmed the high expression of prognostic genes in LUAD. Importantly, *PPP1R14B* expression was particularly significant, and it potentially influenced the LUAD malignant phenotype.

**Conclusion:**

This study constructed and validated a risk model for LUAD associated with parthanatos, providing new insights into the pathological mechanisms of LUAD and highlighting potential therapeutic targets.

## 1 Introduction

Lung cancer (LC) remains the leading cause of cancer-related mortality globally (1). Its incidence and mortality rates continue to rise annually. Statistics indicate that approximately 2.2 million people are diagnosed with LC annually, with 75% of patients dying within 5 years of diagnosis, representing a significant global health burden (2, 3). Histologically, LC is classified as small cell lung cancer or non-small cell lung cancer (NSCLC). NSCLC includes lung adenocarcinoma (LUAD), lung squamous cell carcinoma, and large cell carcinoma, with LUAD being the most common subtype, accounting for 40%–45% of all LC cases (4). Although certain advances have been made in the treatment of LUAD in recent years, the overall survival (OS) of patients remains poor, and they often experience tumor recurrence and unfavorable prognosis (5). Therefore, gaining deeper insights into the underlying mechanisms of LUAD and identifying novel biomarkers are crucial for predicting patient prognosis and developing personalized treatment strategies.

Parthanatos, as a poly(ADP-ribose) polymerase-1 (PARP-1)–dependent cell death process (6), represents a distinct form of programmed cell death from apoptosis, necrosis, and other cell death modalities. Concerning the specific mechanism of parthanatos, DNA damage triggers PARP-1 hyperactivation, leading to the accumulation of poly(ADP-ribose) polymers. This results in mitochondrial membrane depolarization and the translocation of apoptosis-inducing factor (AIF) from the mitochondria to the nucleus. Within the nucleus, AIF helps activate the nuclease macrophage migration inhibitory factor (*MIF*), which selectively cleaves single-stranded DNA, ultimately causing cell death.

Tumorigenesis is a complex process driven by exogenous and endogenous stimuli, including endogenous ROS-mediated DNA backbone damage. PARP-1 is a key enzyme involved in DNA damage repair and recombination, displaying great promise as a drug target for cancers with DNA repair deficiencies. This is consistent with observed increases in PARP-1 expression in bladder cancer cell lines and related tumors (7). PARP-1 inhibitors can selectively kill cancer cells with homologous recombination deficiencies, and they are commonly used for maintenance therapy or in the treatment of recurrent cancers, such as ovarian, prostate, and breast cancers, demonstrating significant potential to overcome tumor drug resistance (8, 9). However, reports on PARP-1 or parthanatos in the context of LUAD are scarce. There is a compelling need to clarify the relationship between parthanatos-related genes (PARGs) and LUAD to provide new insights for targeted therapy.

Therefore, our study collected transcriptomic and single-cell RNA sequencing (scRNA-seq) data from public databases. Leveraging 101 machine learning algorithms, we identified parthanatos-related biomarkers. Through a series of bioinformatics approaches, we systematically investigated the mechanisms by which these biomarkers influence the prognosis of patients with LUAD, aiming to provide new perspectives for LUAD treatment.

## 2 Materials and methods

### 2.1 Data acquisition

Transcriptomic and corresponding clinical data for LUAD were obtained from The Cancer Genome Atlas (TCGA) database (https://portal.gdc.cancer.gov/). After removing samples lacking survival information, the TCGA-LUAD training set included 511 tumor samples and 59 normal tissue samples. Data from the Gene Expression Omnibus database (https://www.ncbi.nlm.nih.gov/) were used for validation. The GSE31210 dataset (226 LUAD samples with survival data) served as the testing set, and the GSE72094 dataset, based on the GPL15048 platform, included a total of 422 LUAD samples, among which 398 samples had complete survival data. Additionally, the GSE131907 scRNA-seq dataset (11 LUAD and 11 paired normal samples) was utilized for further analysis. Nine PARGs have been described in the literature (10), namely *PARP* (*PARP1*), *MIF*, *AIFM1*, *HSP70* (*HSPA4*), *PAAN*, *ARH3*, *RNF146*, *ADPRHL2*, and *OGG1*.

### 2.2 Differential expression analysis

DESeq2 package (v 3.50.3) (11) was employed to identify differentially expressed genes (DEGs) between LUAD and control samples within the TCGA-LUAD dataset (|log_2_ fold change [FC]| > 1 and p.adj< 0.05). Based on log_2_FC, the 10 most upregulated and downregulated genes were established and ranked in descending order, and they were subsequently highlighted in a volcano plot using the ggplot2 package (v 3.3.6) (12). A heatmap was constructed using the ComplexHeatmap package (v 2.14.0) (13).

### 2.3 Identification of candidate genes

Using the TCGA-LUAD dataset and nine PARGs, ssGSEA scores were calculated for each sample using the GSVA package (v 1.42.0) (14). An optimal cutoff stratified samples into high- and low-score groups. Prognostic differences were assessed by Kaplan–Meier (K–M) curves analysis. Weighted gene co-expression network analysis (WGCNA) was then performed using the WGCNA package (v 1.71) (15). After removing outliers, a soft-thresholding power was selected to achieve a scale-free network (R² = 0.85). An adjacency matrix, a topological overlap matrix, and gene modules were subsequently constructed (minModuleSize = 50, mergeCutHeight = 0.25). Key modules significantly correlated (|correlation coefficient [cor]| > 0.3, p< 0.05) with the PARG scores were identified. Candidate genes were derived by intersecting DEGs with genes from these key module genes.

### 2.4 Construction and validation of the risk model

Candidate genes within TCGA-LUAD were assessed by univariate Cox regression and the proportional hazards (PH) assumption test *via* the survival package (v 3.5-7) (16) (hazard ratio ≠ 1 and p< 0.01). Ten machine learning methods, namely Cox partial least squares regression, stepwise Cox proportional hazards model, random survival forest (RSF), elastic net, survival support vector machine, least absolute shrinkage and selection operator, supervised principal components (PCs), ridge regression, generalized boosted regression modeling, and CoxBoost, were used to create 101 algorithm combinations within a leave-one-out cross-validation outline. The model with the highest concordance index (C-index) was selected to identify prognostic genes. The risk model was derived from prognostic genes, and risk scores for each sample were determined using the predict function. Specifically, risk scores were calculated using the following formula:


$$\text{risk score}=\sum_{i=1}^{n}\text{coef}\;\left(\text{i}\right)\times \exp\text{r}\left(\text{i}\right).$$

In the TCGA-LUAD, GSE31210 and GSE72094 datasets, each patient’s risk score was generated using the risk score formula, and LUAD samples were split into risk cohorts based on the median risk score. Using the survminer package (v 0.4.9) (17), K–M curves were plotted for these groups, and receiver operating characteristic (ROC) curves (1-, 3-, and 5-year) were plotted using survivalROC (v 1.0.3) (18). A higher area under the curve (AUC) indicated greater predictive accuracy.

### 2.5 Construction of the nomogram

A nomogram for predicting 1-, 3-, and 5-year survival for patients with LUAD was constructed using the rms package (v 6.3-0) (19). Higher total scores on the nomogram corresponded to lower survival rates. The nomogram’s predictive accuracy was evaluated using calibration and ROC curves (1-, 3-, and 5-year, via the survivalROC package [v 1.0.3] (18)), with higher AUCs indicating better performance. Furthermore, the ggDCA package (v 1.2, https://CRAN.R-project.org/package=ggDCA) was used to draw decision curve analysis (DCA) curves for risk scores, as well as T, M, and N stages, in the TCGA-LUAD cohort.

### 2.6 Survival analysis based on clinical characteristics

The TCGA-LUAD cohort was stratified by clinical features (age, sex, N/M/T stages). K–M analysis was performed to compare survival between risk groups within each stratum. The distribution of risk scores across clinical subgroups was assessed using the Wilcoxon test (for two groups) or the Kruskal–Wallis test (for three or more groups), with statistical significance set at p< 0.05.

### 2.7 Gene set enrichment analysis

GSEA was undertaken to study the functional pathways related to prognostic genes in patients with LUAD from the TCGA-LUAD dataset. First, prognostic gene correlations were computed and ranked for GSEA. GSEA was conducted using the clusterProfiler package (v 4.7.1) (20) with the “c2.cp.kegg.v2023.1.Hs.symbols.gmt” background set from the Molecular Signatures Database (https://www.gsea-msigdb.org). The top three positively and negatively highlighted pathways (|normalized enrichment score| > 1, p< 0.05) were visualized.

### 2.8 Immune microenvironment evaluation

Using the ssGSEA algorithm, the GSVA package (version 1.46.0) (14) was employed to assess the enrichment scores for 28 immune cell types in LUAD samples. Correlation analysis among immune cells *via* the Wilcoxon test (p< 0.05) and among these cells and prognostic genes (|cor| > 0.3, p< 0.05) was conducted using the psych package (v 2.2.9) (21).

Stromal, immune, and ESTIMATE scores were determined by ssGSEA using LUAD sample data, and their differences between risk groups were assessed. The expression of common immune checkpoint genes (*SIGLEC15*, *PDCD1LG2*, *TIGIT*, *LAG3*, *CD274*, *CTLA4*, *HAVCR2*, and *PDCD1*) (22) was determined in patients from TCGA-LUAD to analyze the disparities among the risk groups. The Wilcoxon rank-sum test was then conducted to compare the expression of these genes between the risk groups (p< 0.05).

### 2.9 Tumor mutational burden (TMB) and drug sensitivity analysis and correlation analysis

Somatic mutations in TCGA-LUAD were analyzed using maftools (v 2.18.0) (23). TMB scores for each individual sample were calculated and compared across risk groups using the Wilcoxon test (p< 0.05). The correlation of TMB with risk scores was evaluated using Spearman’s correlation. Furthermore, the pRRophetic package (v 0.5) (24) was utilized to estimate the half-maximal inhibitory concentrations (IC_50_s) of 138 targeted drugs within patients in TCGA-LUAD, followed by comparisons of IC_50_s across different risk groups to evaluate drug efficacy, with lower values indicating greater efficacy (p< 0.05). Furthermore, to investigate the relationship between protein phosphatase 1 regulatory inhibitor subunit 14B (*PPP1R14B*) and the parthanatos pathway, we performed Spearman’s correlation analysis between *PPP1R14B* and core parthanatos pathway genes (*PARP1*, *AIFM1*, and *MIF*) using the psych package (v 2.2.9). Statistical significance was indicated by |cor| > 0.3 and p< 0.05.

### 2.10 scRNA-seq analysis

In the GSE131907 dataset, cells expressing<200 genes and genes expressed in<3 cells were discarded. Additionally, samples with fewer than 300 cells and those with more than 5000 expressed genes and cells containing mitochondrial gene proportions exceeding 10% were removed. The retained data were calibrated using the LogNormalize and FindVariableFeatures functions in the Seurat package (v 5.0.1) (25). After normalization, the top 2000 highly variable genes (HVGs) were selected using the FindVariableFeatures function. Next, these HVGs were subjected to principal component analysis (PCA) using the RunPCA function, and the optimal number of PCs was selected using an elbow plot for clustering. After PCA dimensionality reduction, cells were organized into clusters using the FindNeighbors and FindClusters functions (resolution = 0.4). Subsequently, cell subtypes were annotated and visualized using marker genes (26) to identify different cell types. Finally, the CellChat package (v 1.6.1) (27) was used to analyze cell communication. To identify key cell types, prognostic gene expression across different cell types was visualized, with key cell types selected according to their significant differential gene expression patterns. Pseudotime analysis of subtypes within key cell types were selected using Monocle (v 2.14.0) (28), with cells arranged along developmental trajectories based on pseudotime. Then, gene expression trends were observed along the pseudotime, highlighting dynamic changes in expression as cells progressed through different developmental stages. Copy number variations (CNVs) were inferred using the inferCNV package (v 1.22.0) (29) to identify malignant cells, and the expression patterns of prognostic genes between malignant and nonmalignant epithelial cells were analyzed.

### 2.11 Cell lines and cell culture

Human LUAD cell lines (A549, H1299, H1650, and H838) were obtained from the American Type Culture Collection (Manassas, VA, USA). Cells were cultured in RPMI-1640 medium (Thermo Fisher Scientific, Waltham, MA, USA) supplemented with 10% fetal bovine serum (FBS, Thermo Fisher Scientific) and 1% penicillin–streptomycin (Thermo Fisher Scientific). All cells were maintained in a humidified incubator at 37 °C with 5% CO_2_. The culture medium was refreshed every 2–3 days, and cells were passaged using 0.25% trypsin-EDTA (Thermo Fisher Scientific) upon reaching 80%–90% confluence.

### 2.12 siRNA transfection

Logarithmic-phase A549 and H1299 cells were trypsinized and seeded into six-well plates at densities of 1.5 × 10^5^ and 1.0 × 10^5^ cells/well, respectively. The cells were cultured in 2 mL of complete medium (RPMI-1640 or DMEM supplemented with 10% FBS) at 37°C in a 5% CO_2_ atmosphere for 20 h. Transfection was initiated when cell confluence reached approximately 40%–50%.

The siRNA transfection reagent complexes were prepared using Opti-MEM (Thermo Fisher Scientific) and Lipofectamine 3000 (Thermo Fisher Scientific). Then, 5 µL of siRNA (20 µM, the specific sequences are presented in **Supplementary Table 1**) were diluted in 125 µL of Opti-MEM and incubated for 5 min at room temperature. Then, 5 µL of Lipofectamine 3000 were diluted in 125 µL of Opti-MEM and incubated for 5 min. The diluted siRNA was added dropwise to the diluted Lipofectamine 3000, mixed gently, and incubated for 20 min at room temperature to allow complex formation.

Prior to transfection, the culture medium in the six-well plates was replaced with 1.75 mL of Opti-MEM per well. Then, 250 µL of the siRNA–lipid complex were then added dropwise to each well and distributed evenly *via* gentle agitation. After 6 h of incubation, the transfection medium was replaced with 2 mL of fresh complete medium containing 10% FBS. The cells were further cultured for 72 h and then harvested for subsequent RNA extraction.

### 2.13 Construction and transfection of the overexpression vector

The full-length coding sequence of human *PPP1R14B* (NM_001013628.3) was amplified and cloned into the pcDNA3.1-3×Flag-Puro vector (or pLenti-CMV-MCS-Puro) at the *Bam*HI and *Xho*I restriction sites. The recombinant plasmid was confirmed by Sanger sequencing. An empty vector was used as a negative control. Transfection was performed using Lipofectamine 3000 according to the manufacturer’s instructions.

### 2.14 Cell proliferation assay

Logarithmic-phase cells were enzymatically dissociated and resuspended in complete medium. The cell density was adjusted to 2 × 10^3^ cells/well for both A549 and H1299 cells. The cell suspension (100 µL per well) was seeded into 96-well plates and incubated at 37°C in a 5% CO_2_ atmosphere, and cell growth was monitored at 0, 24, 48, 72, and 96 h.

At each designated time point, the original culture medium was removed. A mixture of 100 µL of fresh medium and 10 µL of CCK-8 reagent (TargetMol, Wellesley Hills, MA, USA) was added to each well, followed by 1.5 h of incubation in the dark. The absorbance (optical density [OD]) at 450 nm was measured using a microplate reader (Thermo Fisher Scientific). The OD was corrected by subtracting the background absorbance of wells containing medium and CCK-8 without cells. The OD at 0 h served as the baseline to calculate the relative proliferation rate. Each experiment was performed in triplicate to ensure reproducibility.

### 2.15 Reverse transcription quantitative polymerase chain reaction

Total RNA was isolated from culture cells or tissue specimens using TRIzol reagent (Thermo Fisher Scientific, 94402). To remove genomic DNA, each RNA sample was treated with DNase I (RNase-free; Thermo Fisher Scientific, 18068015). Each RNA sample was then reverse-transcribed into cDNAs using PrimeScript RT Master Mix (TaKaRa, Shiga, Japan, RR036A). The relative expression of *PPP1R14B*, *MIF*, alpha-1, 3-mannosyltransferase (*ALG3*), chromosome 11 open reading frame 24 (*C11orf24*), and mitotic spindle organizing protein 2A (*MZT2A*) was calculated using the 2^−ΔΔCT^ method. The PCR primers are listed in **Supplementary Table 2**. Each sample was examined at least in triplicate. PCR product specificity was confirmed by melting-curve analysis.

### 2.16 Western blotting

Total cellular proteins were separated by SDS–PAGE and transferred onto a polyvinylidene fluoride (PVDF) membrane (MilliporeSigma, Burlington, MA, USA, ISEQ00010). The PVDF membrane was then incubated with an anti-PPP1R14B antibody (ABclonal, Woburn, MA, USA, A14677) overnight at 4 °C. Target proteins were visualized using ECL Western Blotting Substrate (Thermo Fisher Scientific, 32106).

### 2.17 3-(4, 5-Dimethylthiazol-2-yl)-2, 5-diphenyltetrazolium bromide (MTT) assay

Cell viability was assessed using the MTT assay. Briefly, cells were seeded into 96-well plates at a density of 3 × 10^3^ cells per well and incubated overnight at 37 °C to permit cell attachment. The culture medium was then replaced with fresh medium containing different concentrations. After incubation for 24, 48, 72, or 96 h, 20 µL of MTT solution (5 mg/mL; Sigma-Aldrich, St. Louis, MO, USA) were added to each well, and the plates were incubated for 4 h at 37 °C. Subsequently, the supernatant was carefully removed, and 150 µL of dimethyl sulfoxide were added to dissolve the formazan crystals. The absorbance (OD) at 450 nm was measured using a microplate reader. All experiments were performed in triplicate.

### 2.18 Colony formation assay

For the colony formation assay, cells were digested and seeded into six-well plates at a density of 800 cells per well. The cells were cultured in complete medium at 37 °C with 5% CO_2_ for 14 days, with the medium replaced every 3 days. When macroscopic colonies were visible, the culture was terminated. The cells were washed twice with PBS, fixed with 4% paraformaldehyde for 30 min, and stained with 0.1% crystal violet (Sigma-Aldrich) for 30 min. After washing and drying, colonies containing more than 50 cells were counted manually or using ImageJ software (US National Institutes of Health, Bethesda, MD, USA). All experiments were performed in triplicate.

### 2.19 EdU proliferation assay

Cell proliferation was assessed using the Cell-Light EdU Apollo567 *In Vitro* Kit (RiboBio, Guangzhou, China) according to the manufacturer’s instructions. Briefly, cells were seeded in 24-well plates at a density of 3 × 10^3^ cells per well. After appropriate treatment, cells were incubated with 10 μM EdU-containing medium for 2 h at 37 °C. The cells were then washed with PBS, fixed with 4% paraformaldehyde for 30 min, and permeabilized with 0.5% Triton X-100 for 10 min. Subsequently, the cells were incubated with 1× Apollo staining reaction solution for 30 min in the dark. Nuclei were counterstained with DAPI (or Hoechst 33342) for 5 min to visualize the cells. Images were captured using a fluorescence microscope (IX71, Olympus, Tokyo, Japan). The proliferation rate was calculated as the ratio of EdU-positive cells (red fluorescence) to total DAPI-positive cells (blue fluorescence).

### 2.20 Wound-healing assay

The migration capability of LUAD cells was assessed using a wound-healing assay. Briefly, A549 and H1299 cells were seeded into six-well plates at densities of 5 × 10^5^ and 4 × 10^5^ cells/well, respectively. When the cells reached approximately 90% confluence, two perpendicular scratches (forming a cross shape) were created in the center of each well using a sterile 200-µL pipette tip. The wells were washed three times with PBS to remove detached cells and debris, and the cells were then cultured in serum-free medium. Images of the wounds were captured at the same position at 0 and 48 h using an inverted microscope. The wound closure area was measured using ImageJ software, and the migration rate was calculated as the change in the wound area relative to the initial area at 0 h.

### 2.21 Transwell migration and invasion assays

Cell migration and invasion capacities were assessed using 24-well Transwell chambers with 8-µm pore size polycarbonate membranes (Corning, Corning, NY, USA). For the invasion assay, the upper chambers were precoated with Matrigel (Becton, Dickinson and Company, Franklin Lakes, NJ, USA) diluted 1:8 with serum-free medium, whereas uncoated chambers were used for the migration assay.

Cells were harvested and resuspended in serum-free medium. In total, 3 × 10^4^ A549 cells or 5 × 10^4^ H1299 cells in 200-µL suspensions were seeded into the upper chamber. The lower chamber was filled with 600 µL of medium containing 20% FBS as a chemoattractant. After incubation at 37 °C for 30 h, the nonmigrating or noninvading cells on the upper surface of the membrane were gently removed with cotton swabs. The cells that had migrated or invaded to the lower surface were fixed with 4% paraformaldehyde for 30 min and stained with 0.1% crystal violet for 20 min. After washing and air-drying, the number of cells was counted in at least five randomly selected fields using an inverted microscope.

### 2.22 Poly [ADP-ribose] polymerase 1 (PARP1) Expression by ELISA

The protein expression levels of Human PARP1 were quantified using a commercial Human PARP1 ELISA Kit (FineTest, Wuhan, China; Cat. No. EH1089) based on the sandwich enzyme-linked immunosorbent assay technology, according to the manufacturer’s protocol. Briefly, 100 μL of standard solutions (ranging from 0.625 to 40 ng/mL) or appropriately prepared samples were added into the pre-coated 96-well microplate. The plate was sealed and statically incubated at 37 °C for 90 minutes. Following incubation, the liquid was discarded, and the wells were washed twice with the wash buffer without immersion. Subsequently, 100 μL of biotin-labeled detection antibody working solution was added to each well, and the plate was incubated at 37 °C for 60 minutes, followed by three wash cycles with a 1-minute immersion each time. Next, 100 μL of Horseradish Peroxidase (HRP)-Streptavidin Conjugate (SABC) working solution was introduced, and the plate was incubated at 37 °C for 30 minutes. After five thorough washes with 1-minute immersion per cycle, 90 μL of 3, 3’, 5, 5’-tetramethylbenzidine (TMB) substrate solution was added. The color development was completed in the dark at 37 °C for 10–20 minutes, and the enzymatic reaction was immediately halted by adding 50 μL of Stop Solution. The optical density (OD) of each well was measured at 450 nm using a microplate reader.

### 2.23 Data analysis

The R programming language (v 4.2.2, R Foundation for Statistical Computing, Vienna, Austria) was applied for bioinformatics analyses, with p< 0.05 indicated a statistically significant difference.

## 3 Results

### 3.1 Identification of 49 candidate genes in LUAD

**Figure 1:**
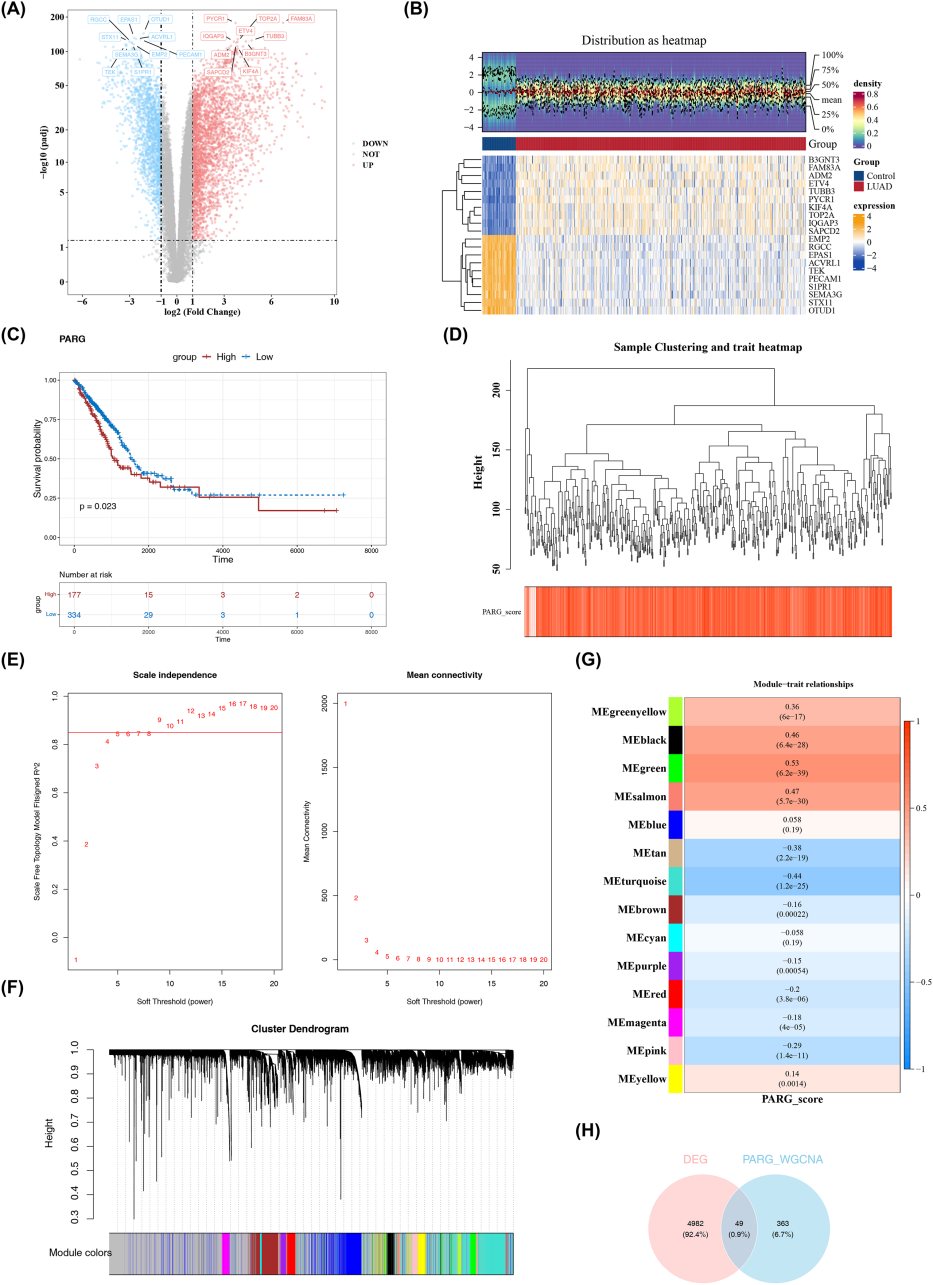
Attainment of 49 candidate genes in LUAD **(A)** The volcano map of DEGs, where red represents upregulation and blue represents downregulation. **(B)** The upper part is a heat map of the expression density of the top 10 genes that are down-regulated, showing the lines of the five quantiles and averages. The next part is the expression heat map of the top 10 genes that are up-and down-regulated in the sample. **(C)** Differences in survival between high and low PARG scores. **(D)** Sample clustering and phenotypic heat map.The branches represent the sample, and the ordinate represents the height of the hierarchical clustering. **(E)** The horizontal axis of the graph represents the power value of the weight parameter, the scale-free fit index on the vertical axis of the left figure, that is, signed R2, the higher the square of the correlation coefficient, the closer the network is to the scaleless distribution, and the vertical axis on the right represents the mean of all gene adjacency functions in the corresponding gene module. **(F)** Module clustering diagram.The upper part is the hierarchical clustering tree map of genes, and the lower part is the gene module. The genes clustered into the same branch are divided into the same module, and different colors represent different modules. **(G)** Heatmap of ssGSEA score correlation between modules and PARS.Darker colors indicate higher correlations. Red is a positive correlation and blue is a negative correlation. Numbers within cells indicate correlation and significance, with the top row being the correlation and the bottom row being the p-value (significance), the left side is the gene module of different colors, and the right color bar represents the correlation range. **(H)** Venn diagram of candidate genes.

The analysis identified 5031 DEGs between the LUAD and control groups, including 3205 upregulated and 1826 downregulated genes in LUAD (**Figures 1A, B**). Based on the ideal boundary for the PARG score (1.431638), patients with LUAD were categorized into high- and low-score groups. K–M analysis identified lower survival in the high PARG score group (p< 0.05; **Figure 1C**). Cluster analysis confirmed the absence of outlier samples (**Figure 1D**), and a soft-thresholding power of 9 was selected to achieve approximate scale-free topology (**Figure 1E**). The dynamic tree cut algorithm revealed 14 distinct gene modules (**Figure 1F**). Among them, the MEgreen module reflected the highest connection with PARG scores (r = 0.53, p< 0.05) and comprised 412 genes (**Figure 1G**; **Supplementary Table 3**), which were selected as key module genes for further analysis. Finally, the intersection of DEGs and key module genes yielded 49 candidate genes (**Figure 1H**).

### 3.2 CoxBoost + RSF model exhibited high predictive accuracy in LUAD

**Figure 2:**
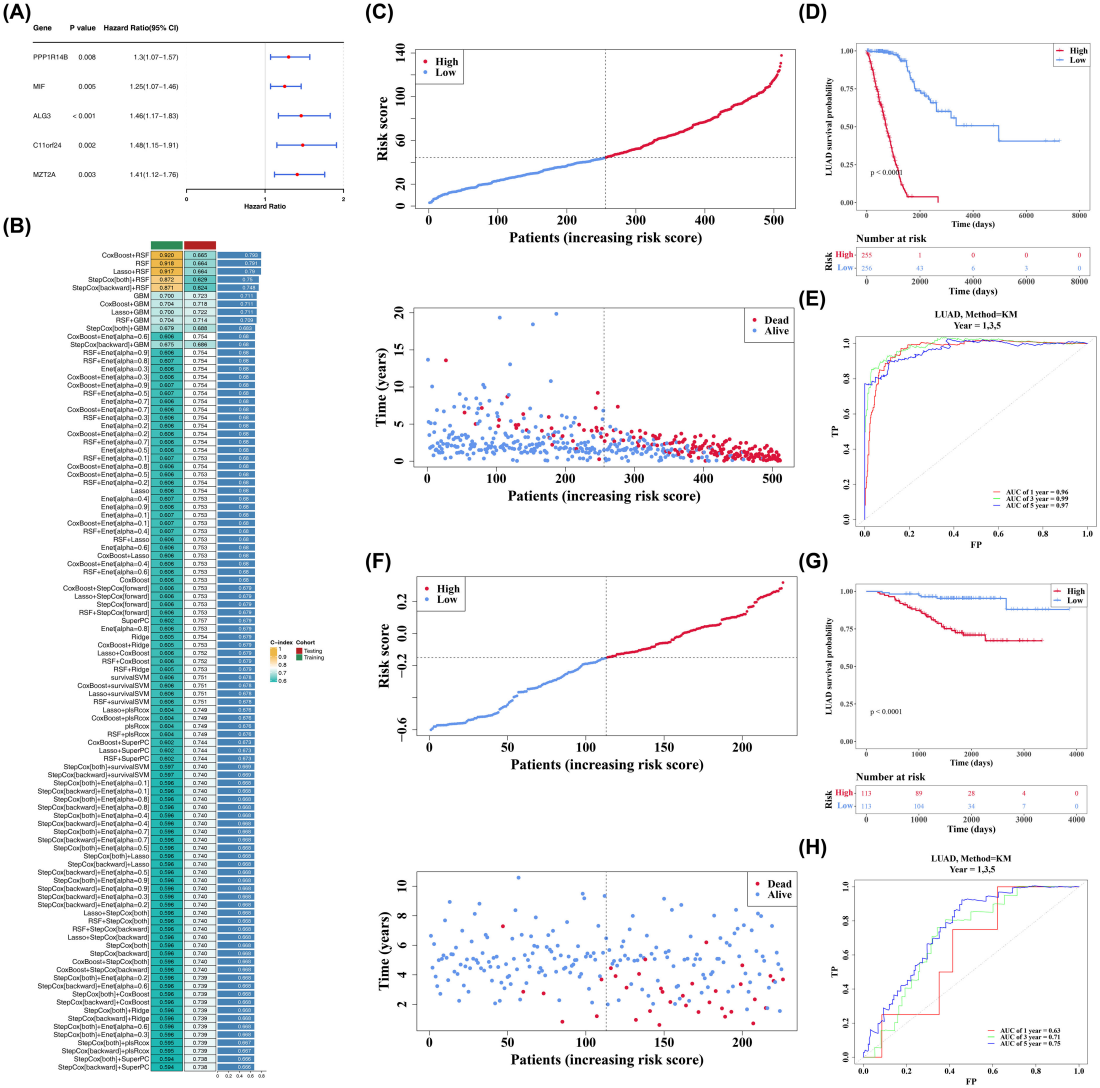
Construction and evaluation of risk models. **(A)** Forest plot of prognostic candidate genes identified by univariate Cox regression. **(B)** 101 machine learning C-index values. **(C)** Top 10 model C index values. **(D)** Risk curve and survival status distribution of TCGA-LUAD samples.The abscissa represents the risk score, and the risk score of patients increases from left to right. Above: red dots indicate high-risk patients, blue dots indicate low-risk patients; Bottom: Red dots indicate dead patients, blue dots indicate surviving patients. **(E)** TCGA-LUAD sample risk model evaluation. Left picture: KM curve, the horizontal axis is the overall survival time (days), and the vertical axis is the survival probability; red is the high-risk group, and blue is the low-risk group. Right: ROC curve. The abscissa is specificity and the ordinate is sensitivity. **(F)** Risk curve and survival status distribution of GSE31210 sample.The abscissa represents the risk score, and the patient’s risk score increases from left to right; upper picture: red dots represent high-risk patients, blue dots represent low-risk patients; bottom picture: red dots represent dead patients, and blue dots represent surviving patients. **(G)** KM curve, the horizontal axis is the overall survival time (days), and the vertical axis is the survival probability; red is the high-risk group, blue is the low-risk group. **(H)** ROC curve, the abscissa is specificity, the ordinate is sensitivity, the area enclosed by the curve and the abscissa is called AUC.

Univariate Cox regression analysis and the PH assumption test identified five prognosis related genes: *PPP1R14B*, *MIF*, *ALG3*, *C11orf24*, and *MZT2A* (**Figure 2A**). The CoxBoost + RSF model demonstrated the best performance among all models, with a C-index exceeding 0.6 within both datasets (**Figure 2B**). As presented in **Supplementary Table 4**, the CoxBoost + RSF model achieved the highest C-index and AUC among the top 10 models, supporting its selection as the optimal prognostic model. The aforementioned five genes were retained in this model as prognosis genes. In the TCGA-LUAD dataset, LUAD samples were split into high- (n = 255) and low-risk groups (n = 256) using the median risk score (**Figure 2C**). A notable survival disparity between the groups was observed in K–M analysis (p< 0.05), with poorer outcomes recorded in the high-risk group (**Figure 2D**). Based on the AUCs at 1 (AUC = 0.96), 3 (AUC = 0.99), and 5 years (AUC = 0.97), the model displayed strong predictive performance (**Figure 2E**). LUAD samples from the GSE31210 set were similarly grouped into high- (n = 113) and low-risk groups (n = 113) according to the median risk score (**Figure 2F**). Consistent with the TCGA-LUAD results, K–M analysis highlighted reduced survival in the high-risk group (p< 0.05; **Figure 2G**). The model maintained a moderate predictive accuracy with AUCs exceeding 0.6 (**Figure 2H**). Furthermore, LUAD samples in the GSE72094 dataset were stratified into high-risk (n=199) and low-risk (n=199) groups based on the median risk score. The number of deaths increased with rising risk scores (**Supplementary Figure 1A**). Kaplan-Meier survival analysis showed that the survival probability of the high-risk group was significantly lower than that of the low-risk group (p=0.021) (**Supplementary Figure 1B**). ROC curve analysis indicated that the 1-year AUC of the risk model was greater than 0.6, and the 3-year and 5-year AUCs were close to 0.6, suggesting the model exhibited limited predictive efficacy in this validation set (**Supplementary Figure 1C**). This outcome might be attributed to differences in sample origin, sequencing platform, ethnic background, and clinical heterogeneity between the GSE72094 dataset and the training cohort.Future studies with larger sample sizes, multi-center designs, and prospective validation will be necessary to further improve the stability and generalizability of the model.

### 3.3 Risk score as a key prognostic factor in the survival nomogram

**Figure 3:**
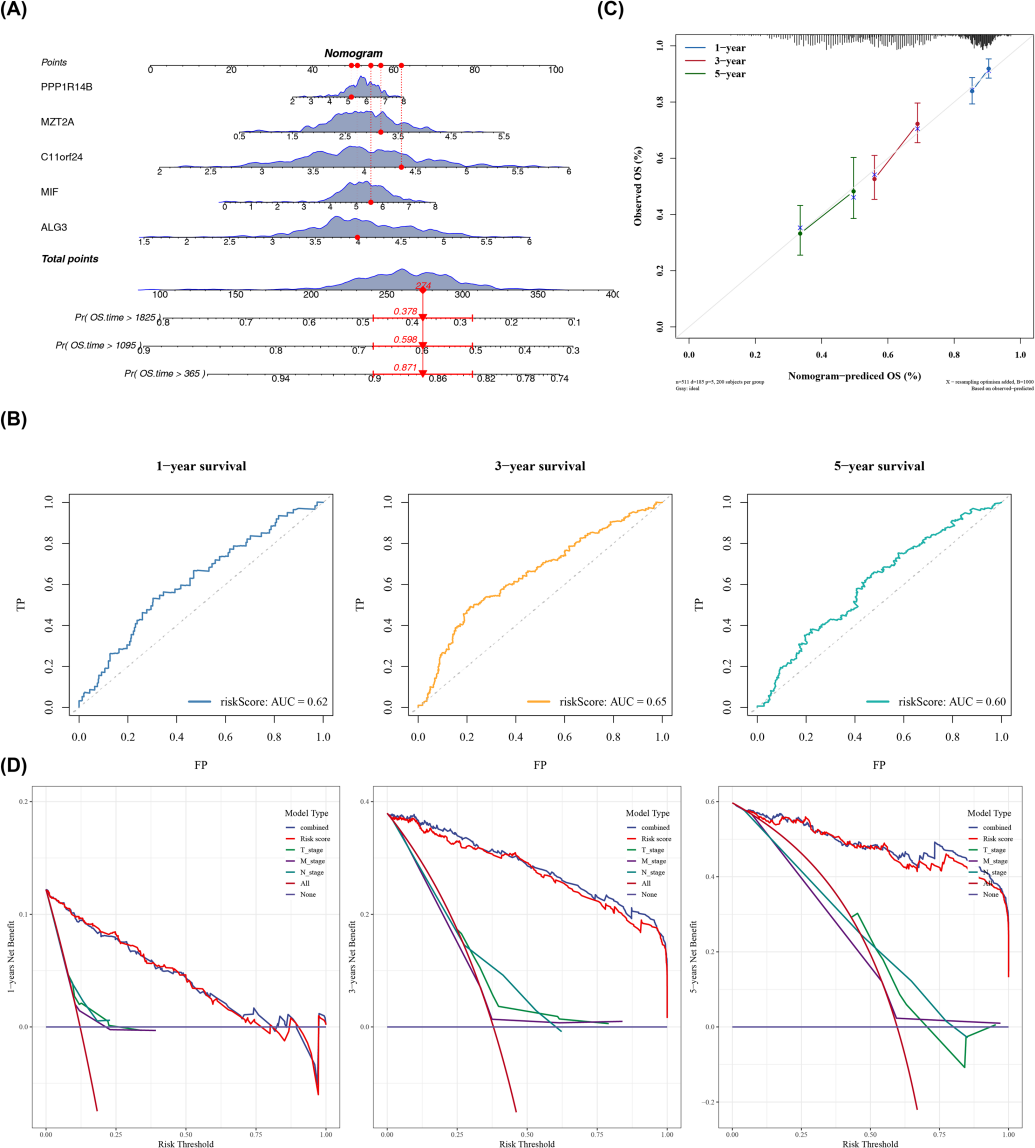
Construction and evaluation of nomogram diagrams. **(A)** Nomogram. **(B)** Nomogram calibration curves. The abscissa is the predicted event rate, and the ordinate is the actual observed event rate, both ranging from 0 to 1. **(C)** Nomogram ROC curve. **(D)** DCA curve analysis.

**Figure 4:**
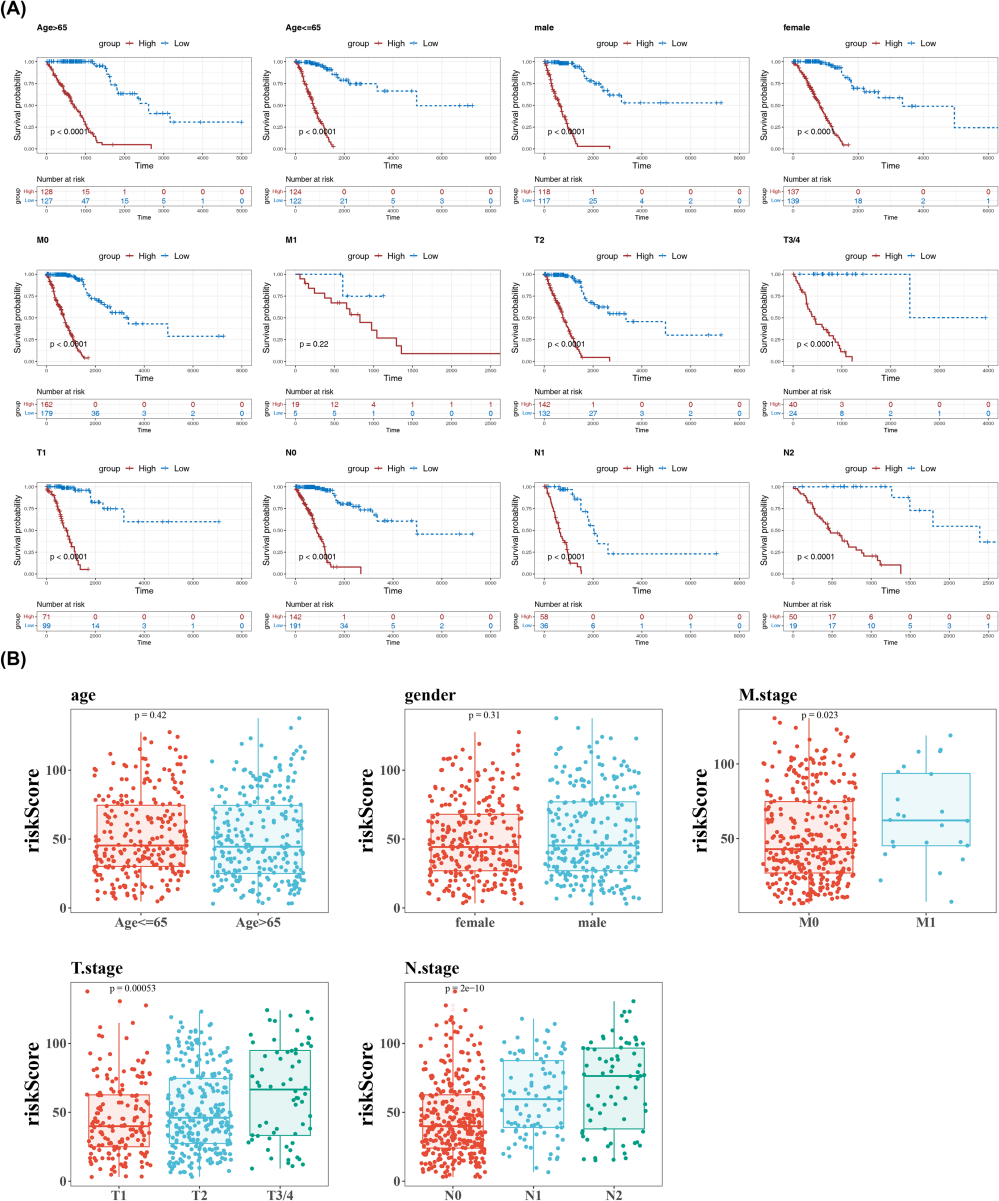
Clinical feature analysis. **(A)** Differences in survival between high and low risk groups among different clinical characteristics groups. **(B)** Risk score differences between groups with different clinical characteristics.

A nomogram was constructed using the five prognostic genes (*PPP1R14B*, *MIF*, *ALG3*, *C11orf24*, and *MZT2A*; **Figure 3A**). The nomogram displayed good predictive accuracy for 1-, 3-, and 5-year OS (AUC > 0.6; **Figure 3B**), although calibration plots indicated declining precision for long-term (5-year) predictions (**Figure 3C**). The results of DCA indicated that the curve for the risk score remained above the “all” and “none” curves and above the curves for the T, M and N stages, indicating that the parthanatos risk score provided a greater net benefit than the conventional TNM staging system in clinical decision-making (**Figure 3D**). K–M curves further validated the risk model across different clinical characteristics. In all subgroups excluding M2, high-risk patients experienced shorter survival (all p< 0.05; **Figure 4A**). Additionally, differential analysis of clinical characteristics revealed that increased risk scores were notably connected to advanced T, N, and M1 stages (all p< 0.05; **Figure 4B**).

### 3.4 Functional characterization of prognostic genes

**Figure 5:**
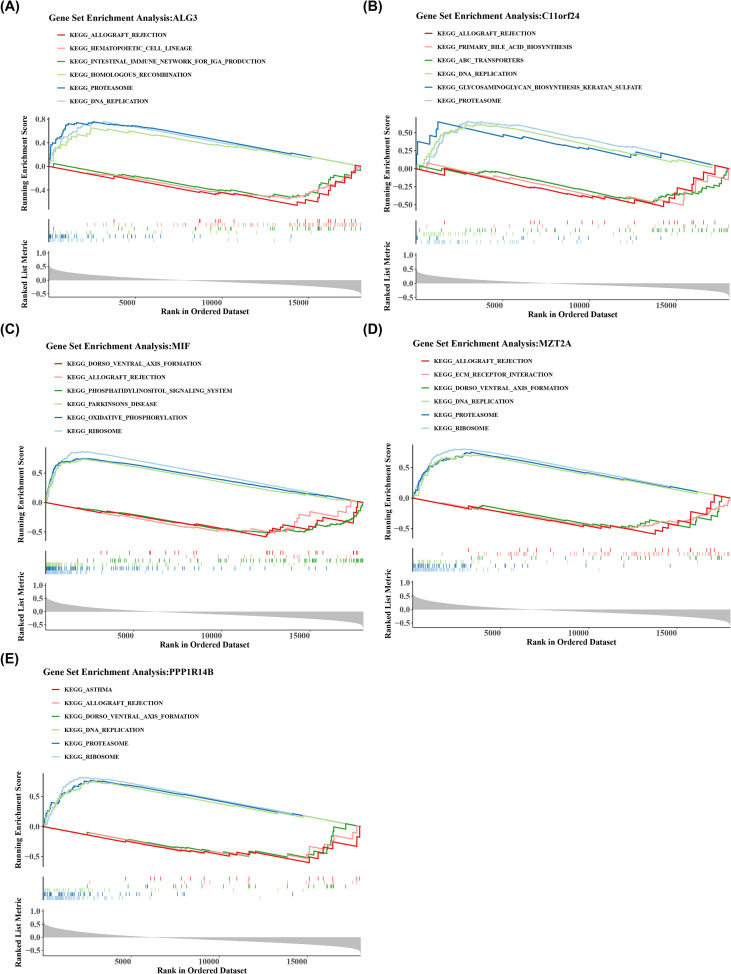
GSEA enrichment analysis of prognostic genes. **(A)** ALG3; **(B)** C11orf24; **(C)** MIF; **(D)** MZT2A; **(E)** PPP1R14B. The figure is divided into three parts. The top part is a line chart of enrichment scores. Each line represents a pathway, and the peak value of each line is the enrichment score of that pathway. The genes before the peak are the core genes in the gene set of that pathway. If the peak is in the upper left corner, it means that the core genes are mainly upregulated genes based on the differential analysis between the high - and low - risk groups. If the peak is in the lower right corner, it means that the core genes are mainly downregulated genes based on the differential analysis between the high - and low - risk groups. The second part uses lines to mark the genes located in the gene set. The third part is the distribution of rank values of all genes.

GSEA revealed significant pathway associations for the prognostic genes. *ALG3* was enriched in 89 pathways, with top positive enrichment in DNA replication, proteasome, and homologous recombination (**Figure 5A**). *C11ORF24* exhibited positive enrichment in DNA replication, proteasome, and glycosaminoglycan biosynthesis and negative enrichment in allograft rejection, primary bile acid biosynthesis, ABC transporters (**Figure 5B**). *MIF* was associated with pathways such as ribosome and oxidative phosphorylation, and it was negatively enriched in dorsoventral axis formation, allograft rejection, and phosphatidylinositol signaling (**Figure 5C**). Both *MZT2A* and *PPP1R14B* were positively enriched in DNA replication, proteasome, and ribosome pathways (**Figures 5D, E**). Multiple genes displayed negative enrichment in allograft rejection. The shared positive enrichment in DNA replication, proteasome, and ribosome pathways underscores the collective importance of the genes in cell growth, protein synthesis, and degradation.

### 3.5 Immune infiltration and immune checkpoint expression between risk groups in LUAD

**Figure 6:**
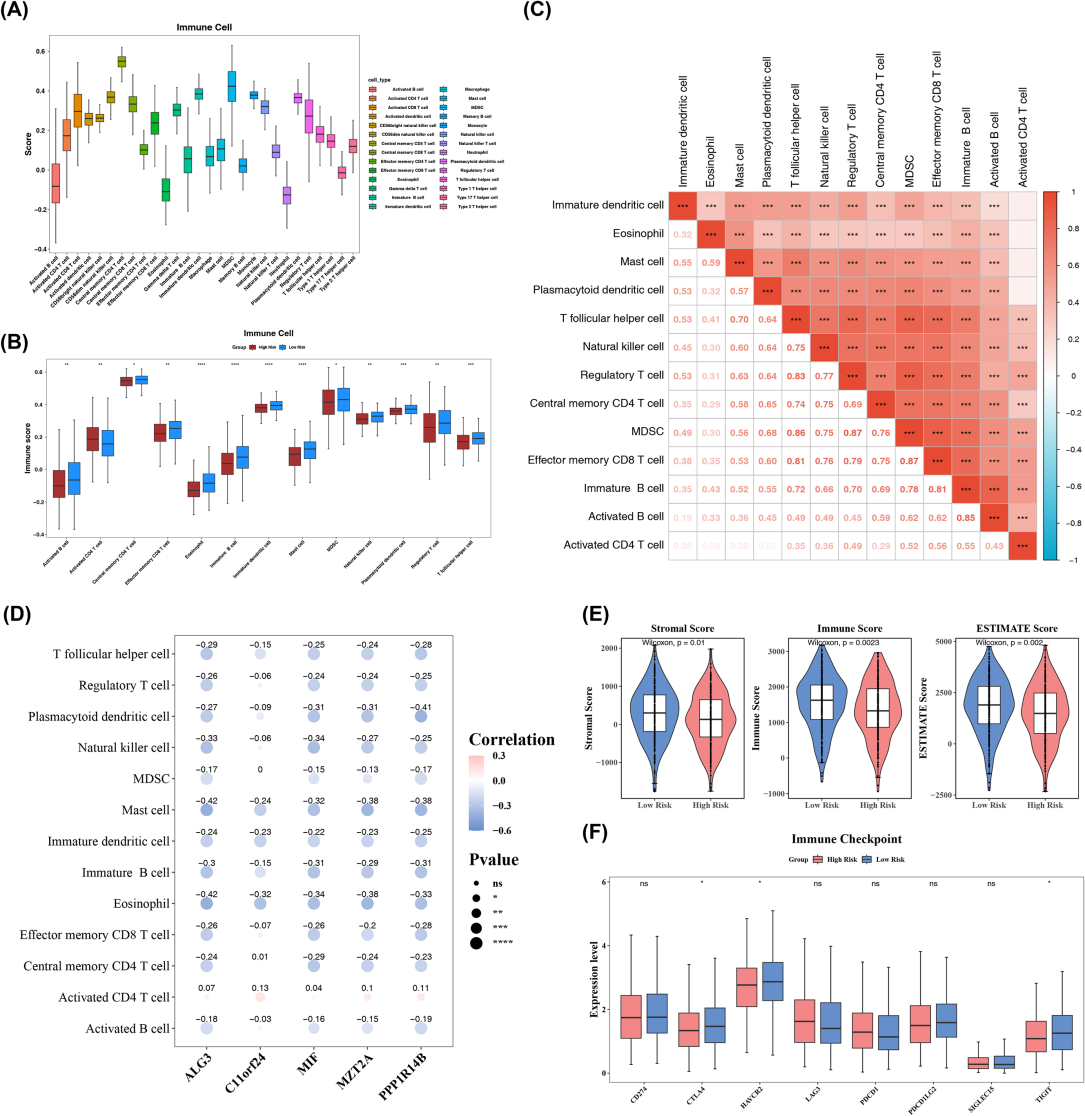
Immune infiltration and immune checkpoint expression between risk groups in LUAD. **(A)** Box plot of immune infiltrating cells. **(B)** Differences in immune infiltrating cells between high and low risk groups. **(C)** Immune infiltrating cell correlation analysis.The abscissa and ordinate coordinates represent differential immune cells; The score represents the size of the correlation coefficient; **(D)** Risk model gene and immune infiltration cell correlation analysis.The abscissa represents the risk model gene, and the ordinate represents the differential immune cells; The score represents correlation, and the circle size indicates significance; **(E)** Differences in ESTIMATE scores between high and low risk groups; **(F)** Significant differences in immune checkpoints in the high and low risk groups.The abscissa represents the immune checkpoint and the ordinate represents the expression level in the sample. ns, not significant; *p<0.05; **p<0.01; ***p<0.001; ****p<0.0001.

Analysis of 28 immune cell types in the TCGA-LUAD cohort revealed distinct distribution patterns between the risk groups (**Figure 6A**). The high-risk group exhibited an increased abundance of activated CD4^+^ T cells, whereas 12 other cell types were enriched in the low-risk group, including regulatory T cells and effector memory CD8 T cells (**Figure 6B**). Most differential immune cells were positively correlated, with the strongest association between myeloid-derived suppressor cells (MDSCs) and effector memory CD8^+^ T cells (cor = 0.87; **Figure 6C**). Notably, all differential immune cells excluding activated CD4^+^ T cells were negatively correlated with the five prognostic genes, with the strongest negative correlation noted between mast cells and *ALG3* (cor = −0.42; **Figure 6D**). Immune, ESTIMATE, and stromal scores were significantly higher in the low-risk group (all p< 0.05; **Figure 6E**), as was the expression of the immune checkpoint genes *CTLA4*, *HAVCR2*, and *TIGIT* (all p< 0.05; **Figure 6F**).

### 3.6 Variations in TMB and drug sensitivity among different risk groups in LUAD

**Figure 7:**
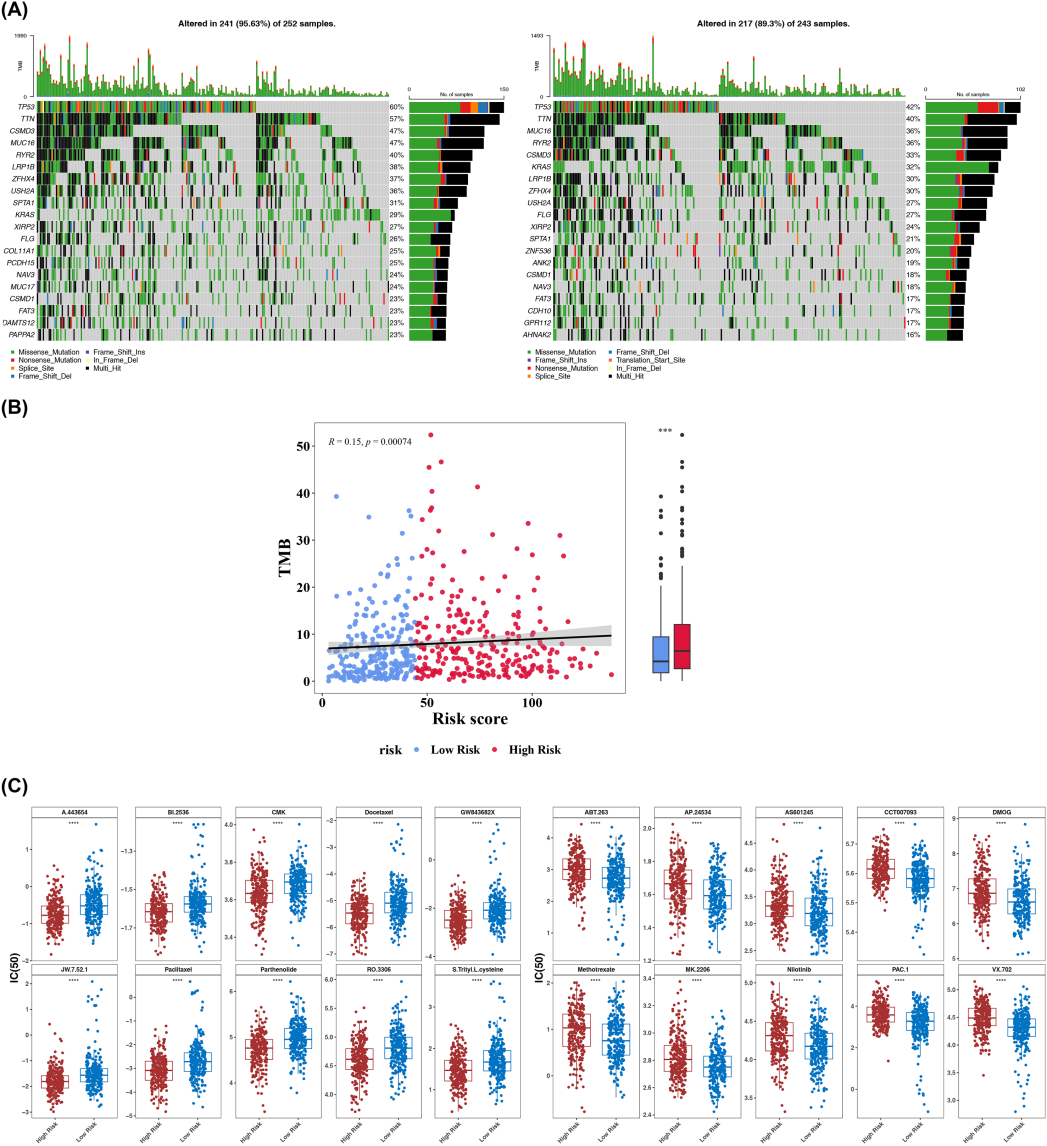
Tumor mutation burden and drug sensitivity analysis. **(A)** Tumor somatic mutations in the high and low risk group (left: high risk; Right: Low risk)The abscissa is the sample, the ordinate is the gene, different colors represent different mutation types, and the histogram is the overall number of mutations for different mutations; **(B)** Scatter plots and box plots of the correlation between TMB and risk score in the high and low risk groups.Blue is the low-risk group, and red is the high-risk group. P is significance and R is correlation. **(C)** IC50 difference between high and low risk groups (left), IC50 difference between high and low risk groups (right).

Analysis of gene mutation frequencies in both risk categories revealed that the top 20 mutated genes primarily exhibited missense and nonsense mutations (**Figure 7A**). Additionally, the high-risk group displayed notably greater TMB than the low-risk group (p< 0.05; **Figure 7B**). Drug sensitivity analysis revealed that 26 drugs, including methotrexate, nilotinib, and VX-702, had significantly higher IC_50_s in the high-risk group, whereas 47 drugs, such as docetaxel, paclitaxel, and parthenolide, had notably lower values in the high-risk group (all p< 0.05). As illustrated in **Figure 7C**, the top 10 drugs exhibited significant IC_50_ differences.

### 3.7 Involvement of key cells in the occurrence of LUAD

**Figure 8:**
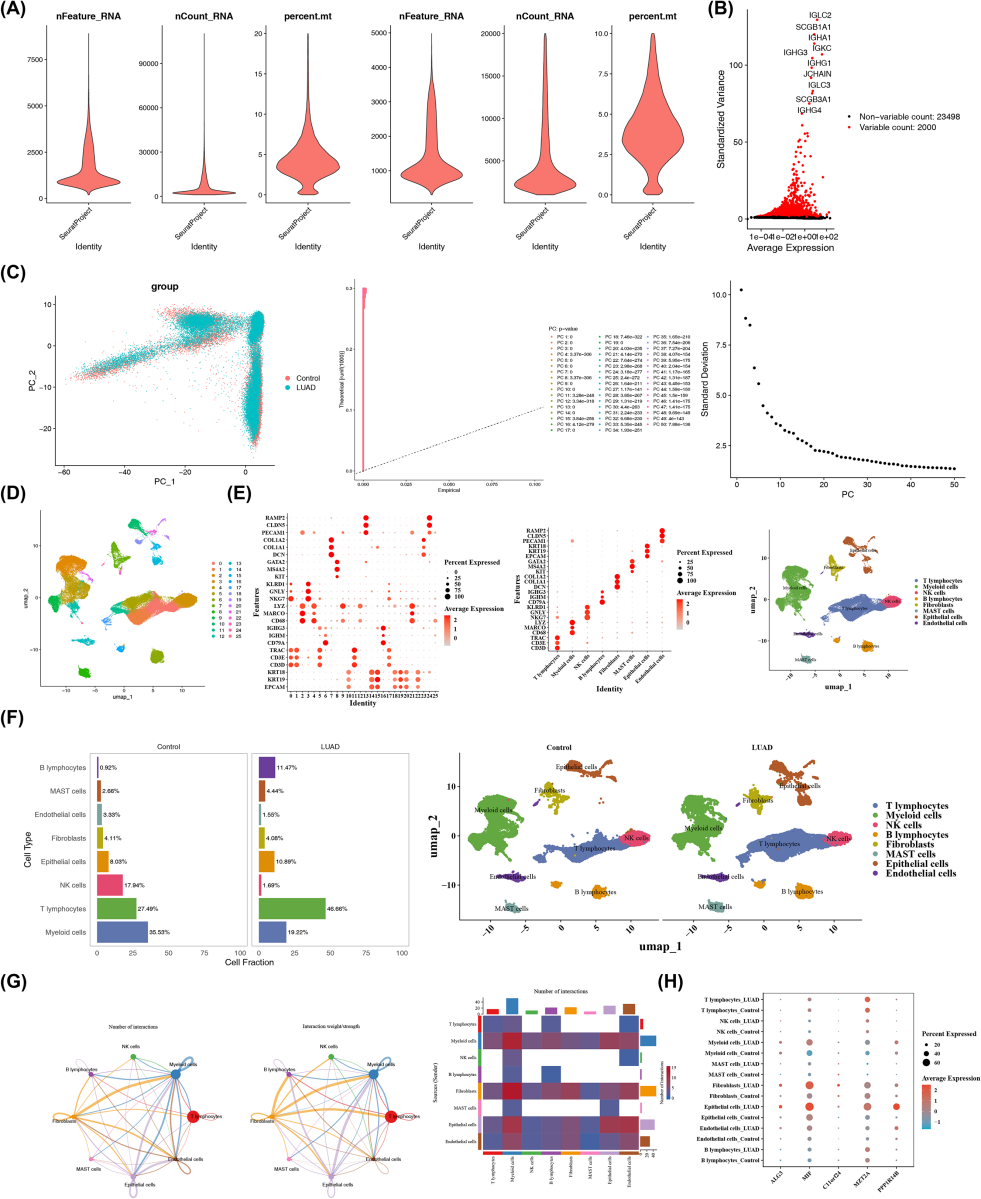
Single - cell analysis. **(A)** Before and after quality control of single - cell data. **(B)** Screening of highly variable genes, with highly variable genes shown in red. **(C)** PCA (Principal Component Analysis) dimensionality reduction plot and scree plot. **(D)** UMAP dimensionality reduction plot of cell clustering, with different colors representing different clusters; **(E)** Expression of marker genes in different cell types and the UMAP dimensionality reduction plot after cell annotation; **(F)** Plot of cell proportions under different groups; **(G)** Diagram of the quantitative relationship of cell - cell communication between different cell types, weight relationship diagram and heatmap; **(F)** Cell scale plot under different groupings; **(G)** Cell communication heatmap; **(H)** The gene is expressed differently in different cell types.

**Figure 9:**
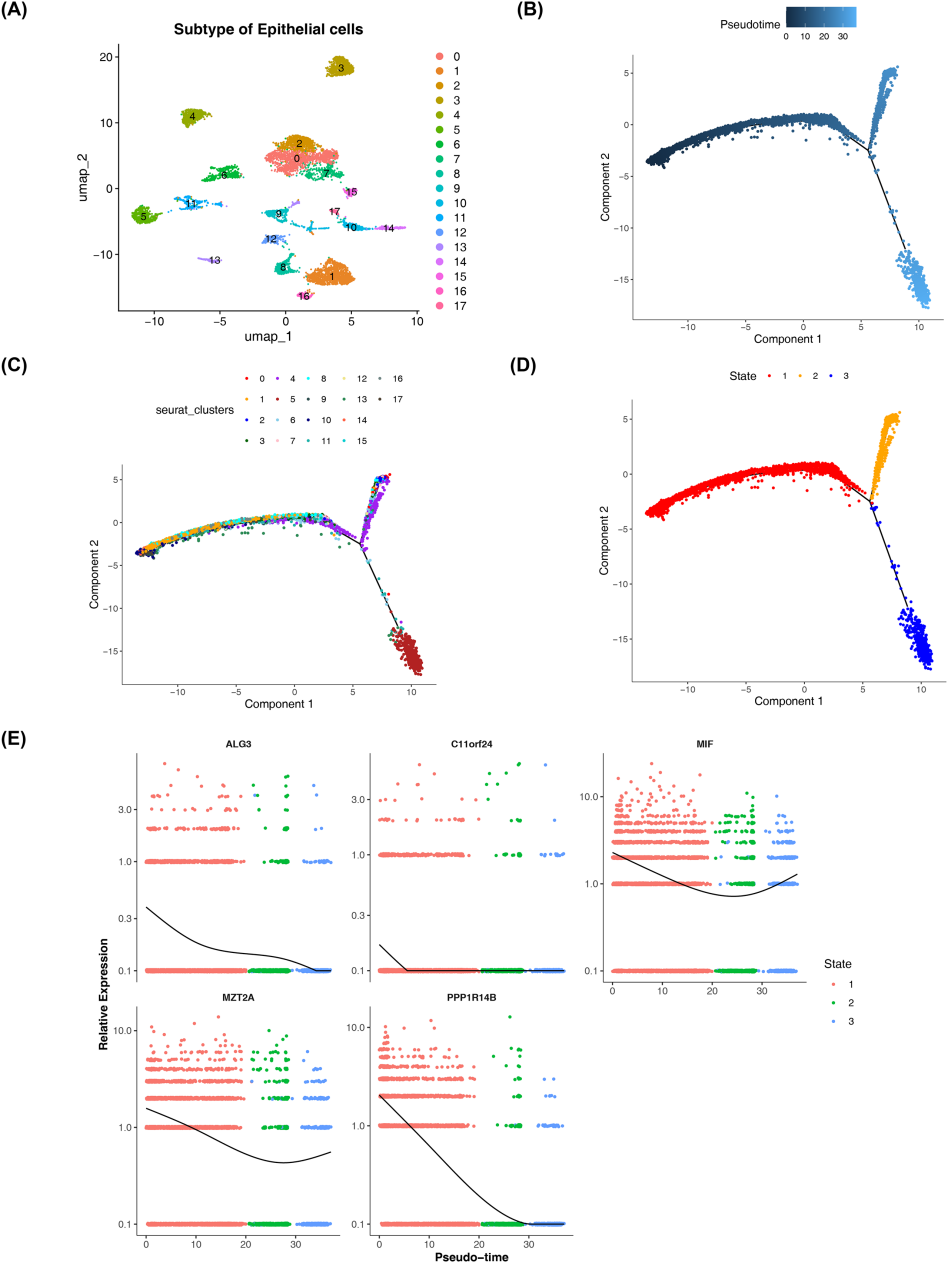
Pseudotemporal analysis of epithelial cells. **(A)** Clustering analysis results of different subpopulations of epithelial cells. **(B)** Pseudotemporal analysis of developmental status over time. **(C)** Trajectory maps of different cell subpopulations. **(D)** Trajectory maps of cell subpopulations at different developmental stages. **(E)** Expression trend diagrams of genes in different developmental stages of epithelial cells.

After quality control, 79, 196 core cells were retained, encompassing 25, 498 genes (**Figure 8A**). Next, 2000 HVGs were identified (**Figure 8B**). PCA was performed, and the top 30 PCs were selected for further analysis (**Figure 8C**). UMAP dimensionality reduction displayed distinct clustering of cell populations (**Figure 8D**), classifying these cells into 26 clusters and annotating eight cell subtypes: T lymphocytes, myeloid cells, natural killer cells, B lymphocytes, fibroblasts, mast cells, epithelial cells, and endothelial cells (**Figure 8E**). In comparison to the control samples, the proportion of T and B lymphocytes was elevated in LUAD, whereas the proportion of myeloid cells was reduced (**Figure 8F**). In further analysis, myeloid cells and fibroblasts exhibited the highest number and strength of cell communications (**Figure 8G**). Subsequently, the expression of the prognostic genes (*PPP1R14B*, *MIF*, *ALG3*, *C11orf24*, and *MZT2A*) across different cell types was analyzed, revealing significant expression differences in epithelial cells between the disease and control groups, designating epithelial cells as the key cell type (**Figure 8H**). In addition, *MIF* was highly expressed in both myeloid cells and fibroblasts, and its expression was significantly increased in the LUAD group. These findings indicated that MIF protein serves as a core secreted ligand mediating crosstalk between myeloid cells and fibroblasts in the LUAD tumor microenvironment, providing a molecular basis for regulating the biological behaviors of these two cell types (**Supplementary Figure 2A**). The number of receptor–ligand interactions was higher in the LUAD group. Specifically, the MIF–(CD74 + CXCR4) pathway interactions between epithelial cells and B lymphocytes, as well as between fibroblasts and B lymphocytes, were most significant in the LUAD group (**Supplementary Figure 2B**). Conversely, the SCGB3A2–MARCO pathway between epithelial cells and myeloid cells was the most significant pathway in the control group (**Supplementary Figure 2C**). Notably, when MIF acted as a ligand, the receptor for all cellular interactions was CD74–CXCR4. UMAP plots revealed high CD74 expression in myeloid cells, indicating that myeloid cells could be the core target cells by which MIF exerts its regulatory effects (**Supplementary Figures 2D, E**). Epithelial cells were further subdivided into 18 subclusters for use in subsequent pseudotime analysis (**Figure 9A**). Finally, analysis of the expression trajectories of the five prognostic genes in epithelial cells indicated that *ALG3*, *C11orf24*, and *PPP1R14B* exhibited decreasing expression across different developmental stages of epithelial cells, whereas *MIF* and *MZT2A* expression initially decreased and then increased (**Figures 9B–E**). These findings provided important insights for further research. CNV analysis revealed that the total number of CNVs was higher in clusters 10, 14, 15, 18, 19, 20, and 22 than in the reference cell clusters (clusters 0, 1, 5, 11, and 17). Therefore, each cluster of epithelial cells could be malignant cells (**Supplementary Figures 3A, B**). The five prognostic genes exhibited significant differences in expression in epithelial cells between the LUAD and control groups (all p< 0.05; **Supplementary Figure 3C**).

### 3.8 Identification of *PPP1R14B* as a crucial oncogene in LUAD

**Figure 10:**
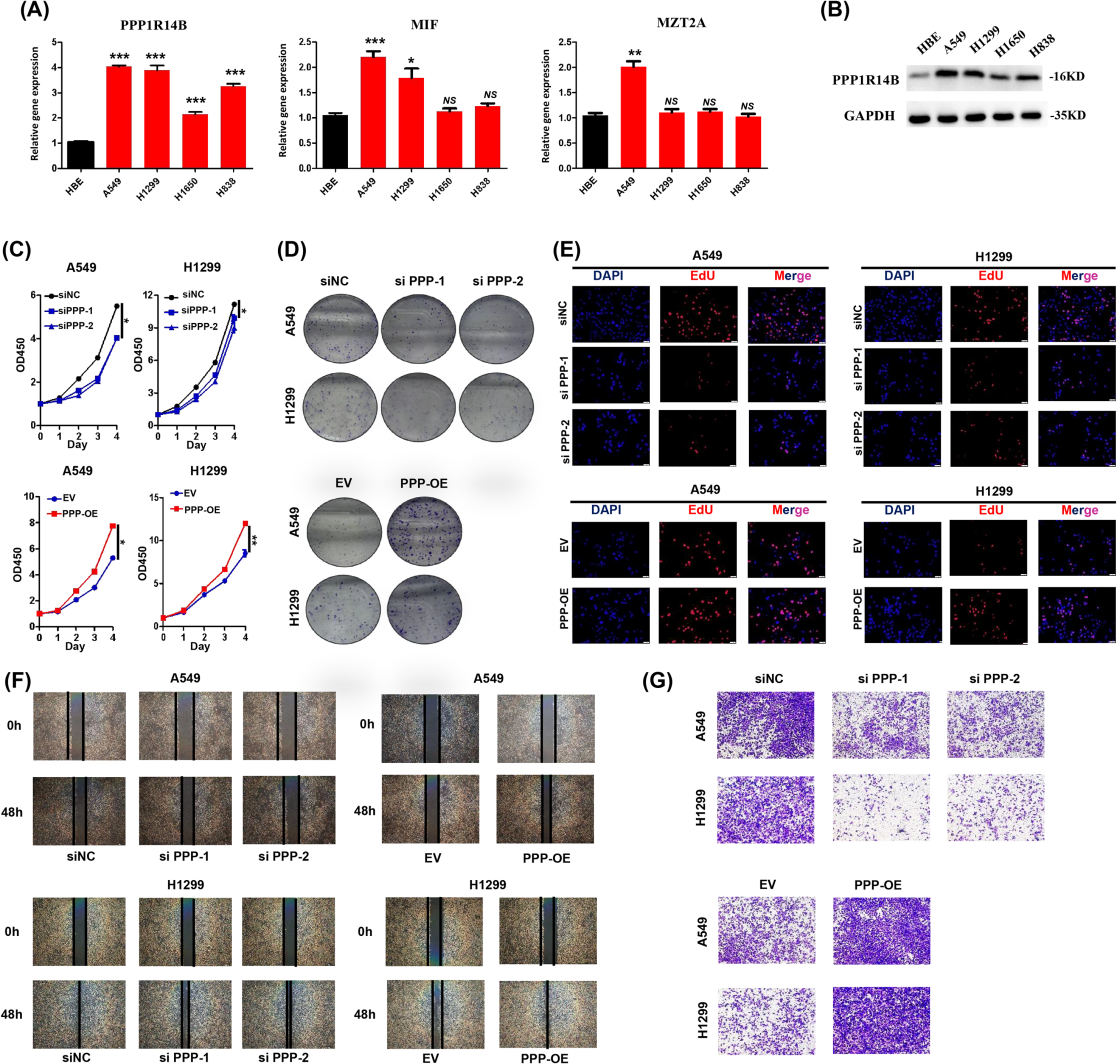
RT-qPCR expression validation analysis. **(A)** Relative *PPP1R14B* expression in LUAD cells; **(B)** PPP1R14B protein expression in LUAD cells; **(C)** Silencing of *PPP1R14B* inhibited MTT of A549 or H1299 cells and overexpressed *PPP1R14B* promoted proliferation of LUAD cells; **(D)** *PPP1R14B* promoted clonogenicity of A549 or H1299 cells; **(E)** Silencing of *PPP1R14B* inhibited EDU of A549 or H1299 cells and overexpressed *PPP1R14B* promoted proliferation of LUAD cells; **(F)** *PPP1R14B* promoted clonogenicity of A549 or H1299 cells of A549 or H1299 cells; **(G)** *PPP1R14B* promoted invasion of A549 or H1299 cells of A549 or H1299 cells.

As previously mentioned, *PPP1R14B*, *MIF*, *ALG3*, *C11orf24*, and *MZT2A* were significantly overexpressed in LUAD, and they might influence tumorigenesis through the PARP signaling pathway. By searching the TCGA and GTEx databases, we found that *PPP1R14B*, *MIF*, and *MZT2A* were highly expressed in tumor tissues (**Supplementary Figure 4A**). Correlation analysis revealed that *PPP1R14B* was significantly and positively correlated with core genes of the parthanatos pathway (*PARP1*, *AIFM1*, and *MIF*), providing preliminary evidence for a potential regulatory relationship between *PPP1R14B* and this pathway (**Supplementary Figure 4B**). To validate the expression of these three genes, we performed RT-qPCR in both LUAD cell lines and tissue samples. The results demonstrated that *PPP1R14B* was markedly upregulated in multiple LC cell lines (**Figure 10A**). Furthermore, transcriptional analysis revealed a significant elevation of *PPP1R14B* expression in LUAD cell lines (**Figure 10B**) Subsequently, we investigated the biological significance of *PPP1R14B* in LUAD through *in vitro* experiments. To assess its functional impact, we both silenced and overexpressed *PPP1R14B* in LUAD cells. The results revealed that *PPP1R14B* knockdown significantly suppressed the proliferation of LUAD cells (all p< 0.01), whereas exogenous expression of *PPP1R14B* markedly enhanced cell viability (both p< 0.001; **Figures 10C–E**). Furthermore, silencing *PPP1R14B* consistently reduced the colony formation, invasion, and metastatic capabilities of LUAD cells (all p< 0.01). Contrarily, LUAD cells overexpressing *PPP1R14B* exhibited enhanced colony formation, invasion, and metastasis (all p< 0.001; **Figures 10F, G**). A positive correlation was observed between *PPP1R14B* expression levels and PARP1 enzymatic activity, as evidenced by a significant elevation following *PPP1R14B* overexpression and a pronounced reduction upon its suppression. (both p< 0.05; **Supplementary Figure 5A**)Notably, our findings uncover a role for *PPP1R14B* as a key regulator of AIFM1 nucleocytoplasmic translocation, a pivotal event in the PARP1-mediated signaling cascade. Manipulation of *PPP1R14B* expression directly altered the subcellular distribution of AIFM1: its overexpression promoted AIFM1 nuclear import, whereas its knockdown resulted in AIFM1 retention within the cytoplasm(**Supplementary Figure 5B**). This regulatory effect of *PPP1R14B* on AIFM1 localization further corroborates that PPP1R14B promotes cancer progression specifically through the PARP1 signaling pathway.Taken together, these findings elucidate the oncogenic role of *PPP1R14B* in LUAD.

## 4 Discussion

As parthanatos is a unique form of PARP-1–dependent cell death, its role in tumor progression has attracted increasing attention (31), but its role in LUAD has been insufficiently studied (32, 33). Using the TCGA-LUAD dataset, a robust parthanatos-related prognostic risk model was successfully constructed and verified by integrating differential expression analysis, WGCNA, and various machine learning algorithms. The core findings were that the risk score was an independent prognostic factor of LUAD, and key genes (*PPP1R14B*, *MIF*, *ALG3*, *C11orf24*, and *MZT2A*) were significantly enriched in functional pathways such as DNA replication and proteasomes and associated with changes in the immune microenvironment. Single-cell analysis further revealed the specific expression dynamics of the genes in malignant epithelial cells. *In vitro* experiments confirmed that the significant upregulation of *PPP1R14B* can promote the proliferation, clonal formation, invasion and metastasis of cancer cells, suggesting that it plays a key role in LUAD progression.

Unlike ferroptosis and cuproptosis, which are predominantly characterized by metabolic dysregulation, parthanatos represents a unique PARP-1-dependent form of DNA damage-triggered cell death. This modality is specifically driven by excessive PARP-1 hyperactivation that induces cellular energy depletion and subsequent AIF-mediated extensive DNA fragmentation. In this study, we innovatively integrated the parthanatos signaling axis into the prognostic evaluation system for LUAD.Rather than directly applying well-established canonical cell death gene signatures, we performed unbiased transcriptome-wide screening. Weighted gene WGCNA was utilized to screen module genes tightly associated with parthanatos activity scores. Combined with cross-validation via 101 machine learning algorithms, five non-canonical parthanatos regulators, including PPP1R14B, were finally identified. As a regulatory inhibitory subunit of PP1, *PPP1R14B* facilitates oncogenic progression and establishes a positive feedback loop with the PARP-1/AIF signaling cascade. This novel mechanistic insight overturns the conventional linear paradigm that cell death-related genes invariably exert tumor-suppressive functions. Notably, single-cell transcriptomic analysis further demonstrated that the MIF-CD74-CXCR4 axis mediates aberrant intercellular crosstalk between myeloid cells and cancer-associated fibroblasts in the LUAD microenvironment. Intriguingly, we identified a distinctive immune paradox in the high-risk LUAD subgroup, wherein elevated TMB was accompanied by dampened expression of immune checkpoint molecules. This phenomenon indicates that parthanatos-based molecular signatures can serve as a robust biomarker for guiding individualized immunotherapy. Specifically, high-risk LUAD patients may derive greater therapeutic benefits from the combination of PARP inhibitors and immune infiltration-augmenting strategies, rather than single-agent immune checkpoint blockade therapy. Collectively, our findings establish a novel and reliable paradigm for precise stratification and personalized targeted immunotherapy of LUAD.

The carcinogenic mechanisms of the five prognostic genes were reported in previous studies. *PPP1R14B* encodes a regulatory subunit that modulates the activity of protein phosphatase 1 (30). This gene was found to be significantly overexpressed in NSCLC, including LUAD. Elevated *PPP1R14B* expression is associated with advanced tumor stage, lymph node metastasis, and poor prognosis in LUAD. It is suggested that *PPP1R14B* promotes LUAD progression by influencing cell cycle regulation, DNA repair processes, and immune cell infiltration (31). Specifically, its overexpression is linked to the activation of DNA repair mechanisms and increased genomic instability, which might collectively contribute to malignant progression. Furthermore, *PPP1R14B* expression is correlated with a higher TMB in LUAD, reinforcing its potential role in cancer pathogenesis. Our *in vitro* experiments functionally validated the oncogenic role of *PPP1R14B* and its modulatory governance over the parthanatos signaling cascade, yielding preliminary mechanistic corroboration.These findings lay a foundation for subsequent mechanistic studies and potential targeted drug development (30, 31).

*MIF* encodes the core effector molecule in the execution of parthanatos and is responsible for mediating AIF-dependent large-scale DNA fragmentation (32). This study found that *MIF* was highly expressed in LUAD, and its expression was closely related to an advanced tumor stage and poor prognosis. We speculate that *MIF* drives LUAD progression by coordinating the DNA damage response with the protumor inflammatory microenvironment. Specifically, *MIF* functions as a nuclease to execute the parthanatos program, and its secretion as a cytokine in the tumor matrix is also widely involved in the recruitment and reprogramming of immune cells (33). GSEA demonstrated the enrichment of *MIF* in metabolic pathways such as ribosome biosynthesis and oxidative phosphorylation, suggesting that it can reshape the energy metabolism of tumor cells to support their rapid proliferation. Our *in vitro* experiments further confirmed the oncogenic function of *MIF* in LUAD and provided a theoretical basis for its use as a therapeutic target.*ALG3* encodes an endoplasmic reticulum-associated glycosyltransferase that is responsible for catalyzing the early steps of N-linked glycosylation (34). In this study, *ALG3* was significantly upregulated in LUAD tissues, and high *ALG3* expression was associated with shortened patient survival. *ALG3* might interfere with cell adhesion, signal transduction and immune surveillance by affecting the glycosylation modification of important membrane proteins and secreted proteins, ultimately promoting the malignant progression of LUAD (35). Functional enrichment analysis demonstrated that *ALG3* is highly related to DNA replication and proteasome pathways, suggesting its potential role in maintaining genome stability and protein quality control. In addition, *ALG3* expression was negatively correlated with the abundance of various tumor-infiltrating immune cells, especially mast cells, suggesting its involvement in the construction of a unique immunosuppressive microenvironment. Subsequent functional experiments verified the promoting effect of *ALG3* on the malignant phenotype of LUAD cells. The mechanism of action of *C11orf24* in cells mainly involves the Golgi apparatus and its related protein transport and membrane recycling. *C11orf24* localizes to the Golgi and trans-Golgi networks and participates in anterograde transport from the Golgi to the plasma membrane by colocalizing with the small GTPase RAB6 (36). Our data illustrated that *C11orf24* exhibits abnormally high expression in LUAD, and it is an independent risk factor for predicting poor prognosis. We speculate that *C11orf24* might serve as a new regulatory node to promote tumorigenesis by affecting the cell cycle and DNA damage repair network. GSEA strongly suggested that *C11orf24* is involved in regulating DNA replication and proteasome function, which is highly consistent with the characteristics of rapid proliferation and evasion of apoptosis of tumor cells. Its specific expression pattern in malignant epithelial cell subsets at the single-cell level further supported its core function in tumor cell autonomy. Experimental verification confirmed the expression of *C11orf24* and its driving effect on LUAD cell growth.

*MZT2A* encodes a key component of the highly conserved γ-tubulin ring complex, which plays a central role in microtubule nucleation and spindle assembly (37). This study revealed that *MZT2A* is upregulated in LUAD and related to the aggressive characteristics of tumors. We propose that *MZT2A* can drive LUAD evolution by ensuring mitotic fidelity and chromosomal stability or conversely by inducing genomic instability (37). Enrichment analysis demonstrated that *MZT2A* is closely related to proliferation-related pathways such as cell cycle and DNA replication, which is consistent with its basic function in mitosis. Single-cell sequencing analysis revealed a dynamic expression pattern of *MZT2A* in epithelial cell subpopulations that changes with malignant progression, suggesting that its expression could be closely related to the proliferation status of tumor cells. Preliminary functional experiments provide evidence for the oncogenic potential of *MZT2A* in LUAD. In summary, *PPP1R14B*, *MIF*, *ALG3*, *C11orf24*, and *MZT2A* function synergistically in LUAD. They form a synergistic pathogenic network by jointly regulating core biological processes such as DNA damage repair, cell cycle progression, and tumor immune microenvironment. The collective high expression of these genes jointly promotes the malignant progression of LUAD and leads to poor patient prognosis.

In this study, a robust prognostic risk model was constructed and validated based on five screened prognostic genes for LUAD. Most currently available LUAD prognostic models focus on programmed cell death (PCD) patterns, including apoptosis, pyroptosis, and necroptosis. For example, Li et al. developed a comprehensive PCD-based prognostic model (CPM) that yielded an AUC of 0.735 in the validation cohort (38). Notably, parthanatos represents a unique PARP-1-dependent cell death pathway. As a core regulator of parthanatos, PARP1 is critically involved in DNA damage repair and tumor immune microenvironment remodeling (39), which endows the present model with two prominent superiority over previous PCD-based models. First, the parthanatos signature-based model can reliably reflect tumor DNA repair deficiency and predict the therapeutic sensitivity of PARP inhibitors. A previous meta-analysis has demonstrated that PARP inhibitors significantly improve the overall survival of patients with advanced lung cancer (HR = 0.90, *p* = 0.006) (40), whereas conventional models focusing on apoptosis and pyroptosis fail to incorporate this targeted therapy-related molecular characteristic. Second, parthanatos acts as an immunogenic cell death modality, and its activation efficiently initiates potent antitumor immune responses. Accumulating clinical evidence has indicated that parthanatos-related signatures are strongly correlated with chemotherapy response and clinical survival outcomes in lung cancer patients (41, 42). Accordingly, our model provides a novel and unique perspective for LUAD patient stratification and immunotherapy response prediction, which cannot be achieved by existing prognostic systems.In summary, the newly developed model not only identifies promising prognostic biomarkers for LUAD but also provides a credible theoretical basis for the combined application of PARP inhibitor therapy and immunotherapy, exhibiting great potential for clinical translation.

A nomogram model was constructed using the prognostic genes in this study, and the score was inversely proportional with the 1-, 3-, and 5-year OS probabilities of patients with LUAD. Although the parthanatos-related risk model exhibited excellent predictive performance for 1- and 3-year OS in patients with LUAD, the calibration plot revealed a slight decrease in its predictive accuracy for 5-year OS. This phenomenon is not incidental; instead, it is associated with multiple factors, including heterogeneity in subsequent treatments, spatiotemporal evolution of tumors, and dynamic regulation of related pathways. Following diagnosis and disease progression, patients can receive various interventions such as surgery, radiotherapy, chemotherapy, targeted therapy, and immunotherapy. However, the model in this study was constructed solely based on baseline transcriptomic data without incorporating information related to these late-stage treatments. Variations in the efficacy of different therapeutic regimens can significantly affect patients’ long-term survival. For instance, some patients classified as high risk by the model achieved long-term survival after receiving novel targeted agents or immune checkpoint inhibitor therapy (43, 44), contradicting the high-risk prediction derived from the baseline gene expression profile and consequently reducing calibration accuracy. Meanwhile, spatiotemporal heterogeneous alterations, including clonal evolution, accumulation of gene mutations, and remodeling of the tumor microenvironment, occur during tumor progression. These changes can modify the expression or biological functions of parthanatos-related prognostic genes such as *PPP1R14B*. Furthermore, the oncogenic roles of the genes might be altered by compensatory effects from other signaling pathways or the emergence of drug resistance, thereby compromising the long-term predictive efficacy of models based on baseline gene expression signatures (31, 37, 45).

In addition, the activity of the parthanatos pathway dynamically fluctuates with tumor progression and therapeutic interventions. DNA damage induced by radiotherapy or chemotherapy can activate PARP-1–dependent cell death, which in turn influences long-term prognosis. However, such dynamic effects were not incorporated into model construction (46). Collectively, these factors reflect the complexity of LUAD progression and the dynamic nature of parthanatos pathway regulation, and provide directions for further model optimization. Future studies will incorporate dynamic treatment information, temporal expression of core genes, and characteristics of tumor clonal evolution to further improve the accuracy of long-term survival prediction for patients with LUAD.

Parthanatos is a form of programmed cell death dependent on PARP-1. Its canonical immunological function is to trigger inflammatory responses by releasing damage-associated molecular patterns, thereby initiating antitumor immune responses (47). Immune microenvironment analysis in the present study revealed that immune and stromal scores were significantly decreased in high-risk patients, indicating remarkably insufficient immune infiltration and immunosuppressive characteristics in the tumor microenvironment. Studies have demonstrated that *ALG3* regulates the tumor immune microenvironment, and its elevated expression is closely associated with the infiltration of MDSCs and CD8^+^ T cells (48). *ALG3* can reduce the immunogenicity of tumor cells and impede the recognition and infiltration of immune cells, probably by modulating glycosylation modification of membrane proteins. As a core effector molecule in the Parthanatos pathway, MIF functions both as a nuclease and cytokine. Secreted in its cytokine form, MIF recruits immunosuppressive cells including M2-type tumor-associated macrophages and MDSCs while suppressing the activation of effector memory CD8^+^ T cells, thereby directly shaping an immunosuppressive microenvironment (49, 50). Zheng et al. reported that their high *PPP1R14B* expression group exhibited low immune cell infiltration abundance and low immune scores. *PPP1R14B* inhibits normal immune function or excludes immune cells from infiltrating into the tumor microenvironment by suppressing the IL2–STAT5 signaling pathway (31). This hypothesis was further validated by the correlation heatmap between prognostic genes and immune cells in the present study, which demonstrated that *PPP1R14B* expression was negatively correlated with the abundance of eosinophils, mast cells, and MDSCs. Furthermore, the negative correlations of *C11orf24* and *MZT2A* expression with various immune-infiltrating cells also suggest that these two genes can indirectly reduce the immunogenicity of tumor cells and attenuate the recruitment and activation of immune cells by regulating core biological processes such as DNA replication and the cell cycle. In summary, the five parthanatos-related prognostic genes identified in this study can regulate the tumor immune microenvironment at different levels through their specific functions. These mechanisms interact and act synergistically, ultimately leading to a phenotype characterized by low immune infiltration and high immunosuppression in the tumor microenvironment of high-risk patients. Additionally, this process is coupled with abnormal parthanatos pathway activation, jointly affecting the prognosis of patients with LUAD.

Furthermore, this study uncovered a critical clinical paradox in LUAD: the high-risk subgroup exhibited elevated TMB levels but significantly decreased expression of immune checkpoint molecules, including *CTLA4*, *HAVCR2*, and *TIGIT*. Similar contradictory phenomena have been documented in multiple malignancies, which is primarily attributed to tumor immune escape via “immune ignorance” rather than adaptive immune resistance. Despite the abundant generation of mutation-derived neoantigens, defective antigen presentation fails to initiate effective anti-tumor immune responses (51). Consistent with these findings, the high-risk cohort in our study displayed an increased proportion of activated CD4 T cells, alongside pronounced exhaustion of effector memory CD8 T cells and markedly reduced immune and stromal scores. This phenotype aligns with the “non-T cell inflamed” tumor characteristic proposed by Haibe et al., wherein robust neoantigen burden cannot drive sufficient T cell recruitment due to inherent microenvironmental defects (52). From the perspective of parthanatos signaling, excessive PARP-1 activation induces severe NAD depletion and subsequent cellular energy crisis, which impairs the cGAS-STING innate immune pathway. This dysfunction suppresses the secretion of type I interferons and downstream chemokines, ultimately constructing an “interferon desert” tumor microenvironment (53). This mechanistic pattern is consistent with the immune resistance mechanism of small cell lung cancer (SCLC) revealed by DDR-IF scoring studies: genomic instability alone cannot automatically transform into tumor immunogenicity, and the integrity of innate immune sensing serves as the decisive determinant of immune checkpoint inhibitor (ICI) efficacy (53). Collectively, the immunosuppressive phenotype in the high-risk subgroup is not driven by the hyperactivation of canonical immune checkpoint pathways, but may result from parthanatos signaling aberration-mediated immune infiltration deficiency. This finding yields profound clinical implications for individualized therapeutic stratification. For high-risk LUAD patients, monotherapy targeting *CTLA4* or *TIGIT* may achieve limited efficacy, whereas the combination of PARP inhibitors and immune infiltration-promoting strategies represents a superior therapeutic option. In contrast, the low-risk subgroup is characterized by a “high immune infiltration–high checkpoint expression” phenotype, which confers greater susceptibility to single-agent immune checkpoint blockade therapy.

In addition, multiomics analysis in this study further revealed the profound biological basis of this risk model. Single-cell transcriptome analysis revealed abnormally active cellular communication between myeloid cells and fibroblasts, which echoed the expression patterns of PARGs. We speculate that these key genes can promote the differentiation of myeloid cells to an immunosuppressive phenotype by regulating the secretion of cytokines and chemokines while activating cancer-associated fibroblasts and jointly shaping a microenvironment conducive to tumor growth and metastasis. At the same time, drug susceptibility analysis provided important clues for clinical translation of risk models, and the higher sensitivity to platinum drugs in high-risk patients could be related to their active cell cycle progression and genomic instability (54). The higher TMB in the high-risk group explains its more aggressive clinical features at the genomic level. Together, these findings suggest that parthanatos-related features are prognostic indicators, and they reflect the specific biological behavior of LUAD, providing a molecular basis for future targeted therapy and chemotherapy regimen selection for high-risk patients.

To summarize, we established a robust parthanatos-related prognostic signature for LUAD leveraging integrated multi-omics analysis and machine learning workflows, while initially characterizing the pro-tumorigenic function of the hub gene *PPP1R14B*. Nevertheless, certain limitations persist, warranting further optimization and deeper mechanistic investigation in subsequent research.To address the aforementioned deficiencies, we have designed rigorous experiments to validate that *PPP1R14B* modulates PARP1 activity, and we propose targeted directions for future investigations. Mechanistically, *in vitro* assays evaluating the impacts of *PPP1R14B* on PARP1 enzymatic activity and AIF nuclear translocation (via immunofluorescence) were implemented to dissect the precise molecular mechanisms by which *PPP1R14B* regulates the PARP1 signaling pathway. Moving forward, we plan to construct *in vivo* cell line-derived xenograft (CDX) and patient-derived xenograft (PDX) LUAD models to validate the oncogenic potential of the signature genes and evaluate their therapeutic responsiveness to PARP-targeted interventions. Moreover, the integration of spatial transcriptomics and large-scale clinical cohorts will facilitate the in-depth elucidation of the molecular mechanisms underlying immune exclusion in high-risk LUAD patients. In terms of model optimization, multi-center prospective clinical data and additional independent external cohorts will be enrolled in future studies. Advanced regularization algorithms and strict cross-validation strategies will be adopted to refine model parameters, mitigate overfitting risks, and enhance the predictive robustness and cross-population generalizability of the signature. Furthermore, comprehensive functional verification of the remaining prognostic genes will be conducted to consolidate the theoretical foundation for the clinical translation of parthanatos-based stratified targeted and immunotherapeutic strategies in LUAD.

## Data Availability

The original contributions presented in the study are included in the article/**Supplementary Material**. Further inquiries can be directed to the corresponding authors.

## Supplementary material

Supplementary Figure 1 Validation of the risk model in the GSE72094 dataset. **(A)** Risk curve and survival status distribution of GSE72094 sample. The abscissa represents the risk score, and the patient’s risk score increases from left to right; upper picture: red dots represent high-risk patients, blue dots represent low-risk patients; bottom picture: red dots represent dead patients, and blue dots represent surviving patients. **(B)** KM curve, the horizontal axis is the overall survival time (days), and the vertical axis is the survival probability; red is the high-risk group, blue is the low-risk group. **(C)** ROC curve, the abscissa is specificity, the ordinate is sensitivity, the area enclosed by the curve and the abscissa is called AUC.

Supplementary Figure 2 Cell-cell communication analysis. **(A)** MIF expression in different cell types. **(B)** Ligand-receptor bubble plot in the LUAD group. **(C)** Ligand-receptor bubble plot in the control group. **(D, E)** Expression of CD74 and CXCR4 in different cell types.

Supplementary Figure 3 CNV analysis. **(A)** Heatmap of inferCNV results. The upper panel shows the reference cells (T cells), and the lower panel shows the target cells (epithelial cells). Red indicates CNV amplification, blue indicates CNV deletion, and darker color represents more obvious CNV alterations. **(B)** Boxplot of inferCNV results. Clusters 0, 1, 5, 11, and 17 represent reference cells (T cells), while Clusters 10, 14, 15, 18, 19, 20, and 22 represent unannotated epithelial cells. **(C)** Expression differences of prognostic genes in epithelial cells.

Supplementary Figure 4 **(A)** Expression of *PPP1R14B*, *MIF*, and *MZT2A* in the TCGA and GTEx databases. **(B)** Correlation analysis of *PPP1R14B* with core genes (PARP1, AIFM1, and MIF) in the Parthanatos pathway.

Supplementary Figure 5 **(A)** Overexpression of *PPP1R14B* significantly promoted PARP1 activity, downregulation of *PPP1R14B* resulted in a significant decrease. **(B)** Overexpression of *PPP1R14B* led to a decrease in AIFM1 expression in the cytoplasm and a concurrent increase in the nucleus(left). Knockdown of PPP1R14B increased cytoplasmic AIFM1 while decreasing its nuclear presence(right).

## Glossary

LC: Lung cancer
SCLC: small cell lung cancer
NSCLC: non-small cell lung cancer
LUAD: lung adenocarcinoma
LUSC: lung squamous cell carcinoma
LCC: large cell carcinoma
ADP-ribose: accumulation of poly
AIF: apoptosis-inducing factor
PARGs: PARthanatos-related genes
TCGA: The Cancer Genome Atlas
GEO: Gene Expression Omnibus
FC: fold change
K-M: Kaplan-Meier
WGCNA: Weighted gene co-expression network analysis
TOM: topological overlap matrix
cor: correlation coefficient
PH: proportional hazards
HR: hazard ratio
RSF: random survival forest
LASSO: least absolute shrinkage and selection operator
LOOCV: leave-one-out cross-validation
C-index: concordance index
AUC: area under the curve
ROC: receiver operating characteristic
GSEA: Gene set enrichment analysis
NES: Normalized Enrichment Score
MSigDB: Molecular Signatures Database
TMB: Tumor mutational burden
scRNA-seq: Single-cell RNA sequencing
HVGs: highly variable genes
PCs: principal components
PCA: principal component analysis
ATCC: American Type Culture Collection
RT-qPCR: Reverse transcription quantitative polymerase chain reaction
PVDF: polyvinylidene fluoride
DMSO: dimethyl sulfoxide
DEGs: Differentially Expressed Genes
OS: overall survival
MDSCs: myeloid-derived suppressor cells
NK: natural killer
PP1: protein phosphatase 1
TMB: tumor mutation burden
MIF: Macrophage Migration Inhibitory Factor.
