# Supplementary figures and images for: Identification and validation of parthanatos-related genes in lung adenocarcinoma and construction of a prognostic risk model
Source: Front Immunol. 2026 Jul 8;17:1806560. doi: 10.3389/fimmu.2026.1806560 (PMC13388751; doi:10.3389/fimmu.2026.1806560)

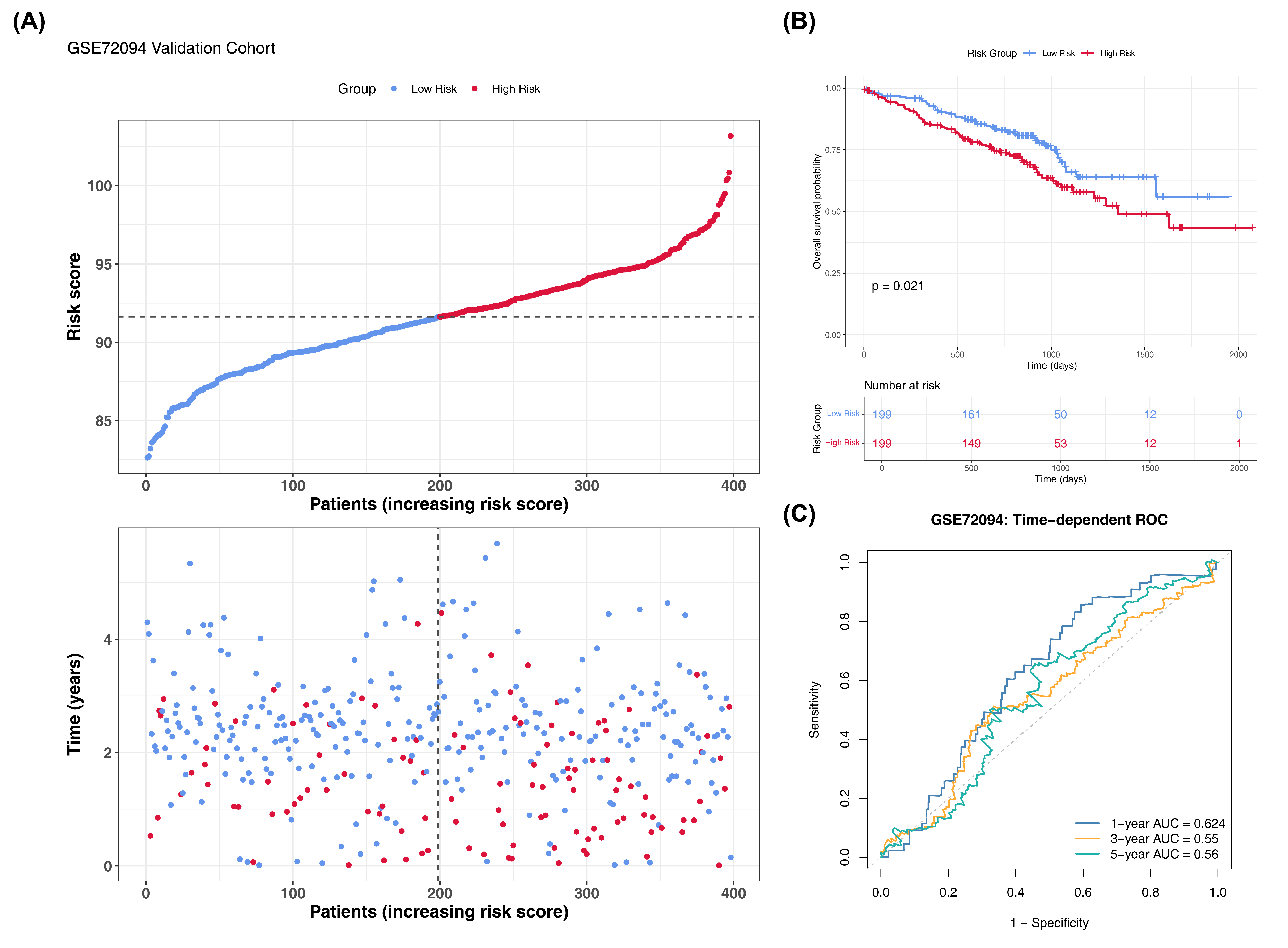

Supplement: Supplementary Figure 1 — Validation of the risk model in the GSE72094 dataset. (A) Risk curve and survival status distribution of GSE72094 sample. The abscissa represents the risk score, and the patient’s risk score increases from left to right; upper picture: red dots represent high-risk patients, blue dots represent low-risk patients; bottom picture: red dots represent dead patients, and blue dots represent surviving patients. (B) KM curve, the horizontal axis is the overall survival time (days), and the vertical axis is the survival probability; red is the high-risk group, blue is the low-risk group. (C) ROC curve, the abscissa is specificity, the ordinate is sensitivity, the area enclosed by the curve and the abscissa is called AUC. [file Image1.tif]

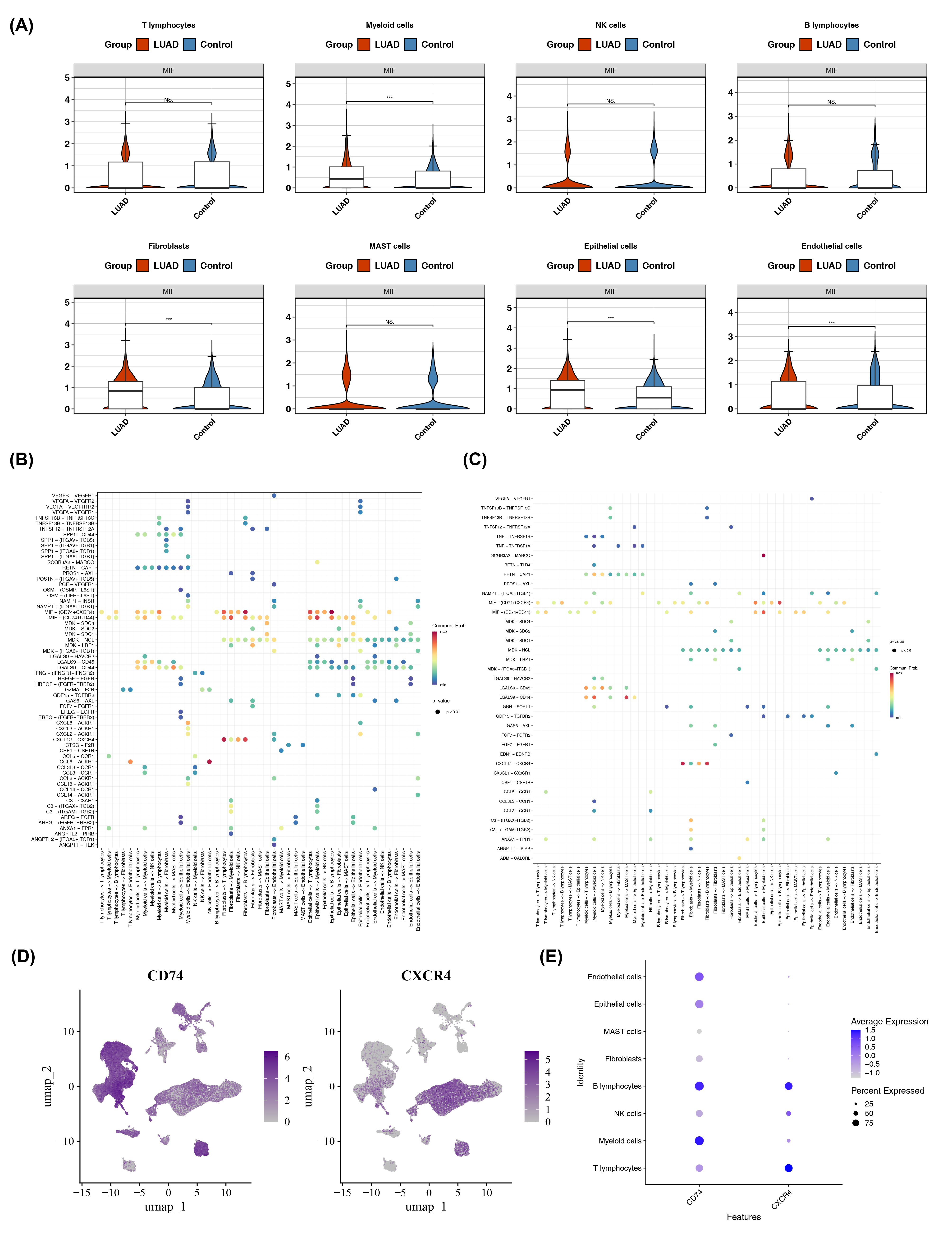

Supplement: Supplementary Figure 2 — Cell-cell communication analysis. (A) MIF expression in different cell types. (B) Ligand-receptor bubble plot in the LUAD group. (C) Ligand-receptor bubble plot in the control group. (D, E) Expression of CD74 and CXCR4 in different cell types. [file Image2.tif]

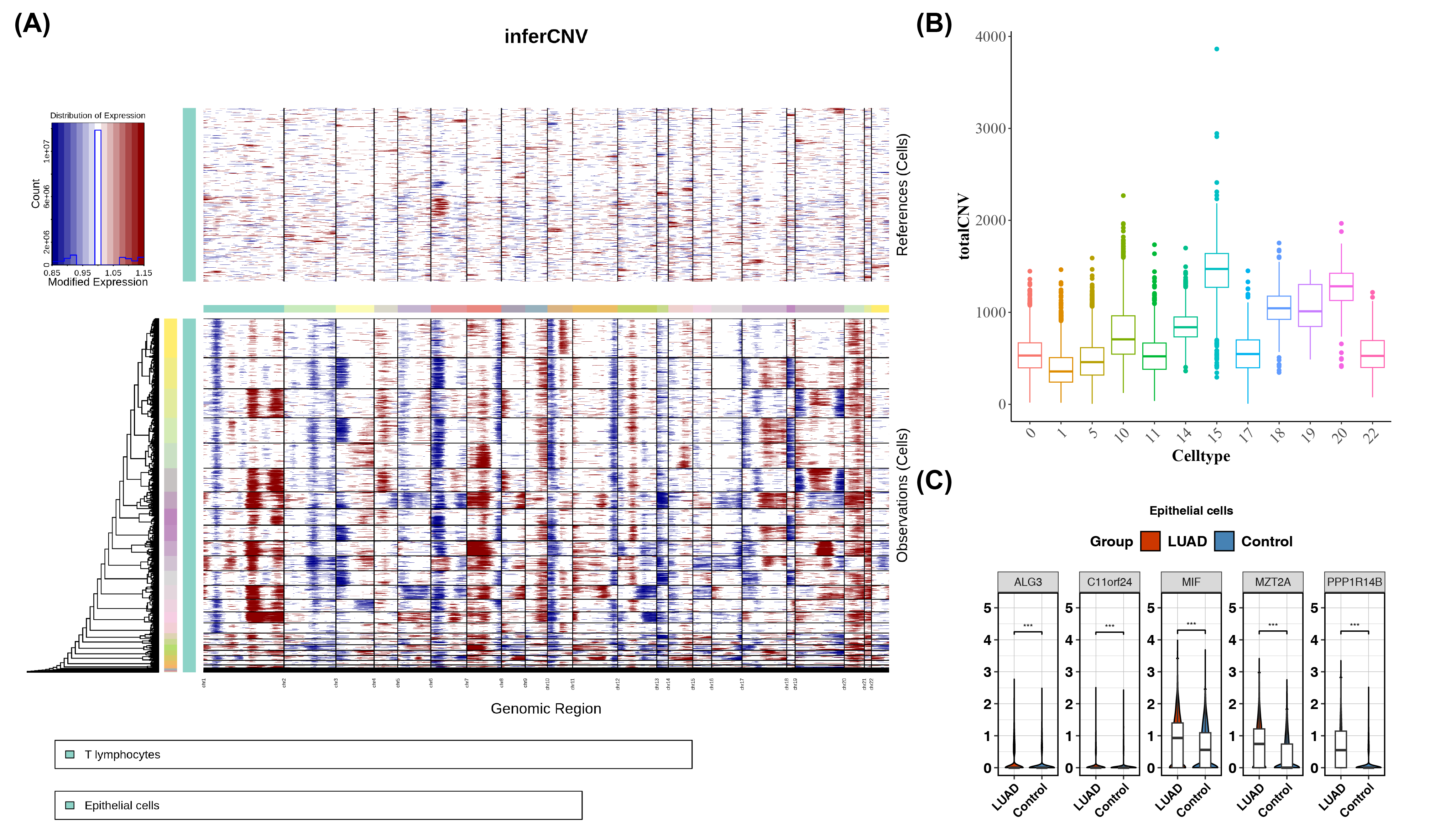

Supplement: Supplementary Figure 3 — CNV analysis. (A) Heatmap of inferCNV results. The upper panel shows the reference cells (T cells), and the lower panel shows the target cells (epithelial cells). Red indicates CNV amplification, blue indicates CNV deletion, and darker color represents more obvious CNV alterations. (B) Boxplot of inferCNV results. Clusters 0, 1, 5, 11, and 17 represent reference cells (T cells), while Clusters 10, 14, 15, 18, 19, 20, and 22 represent unannotated epithelial cells. (C) Expression differences of prognostic genes in epithelial cells. [file Image3.tif]

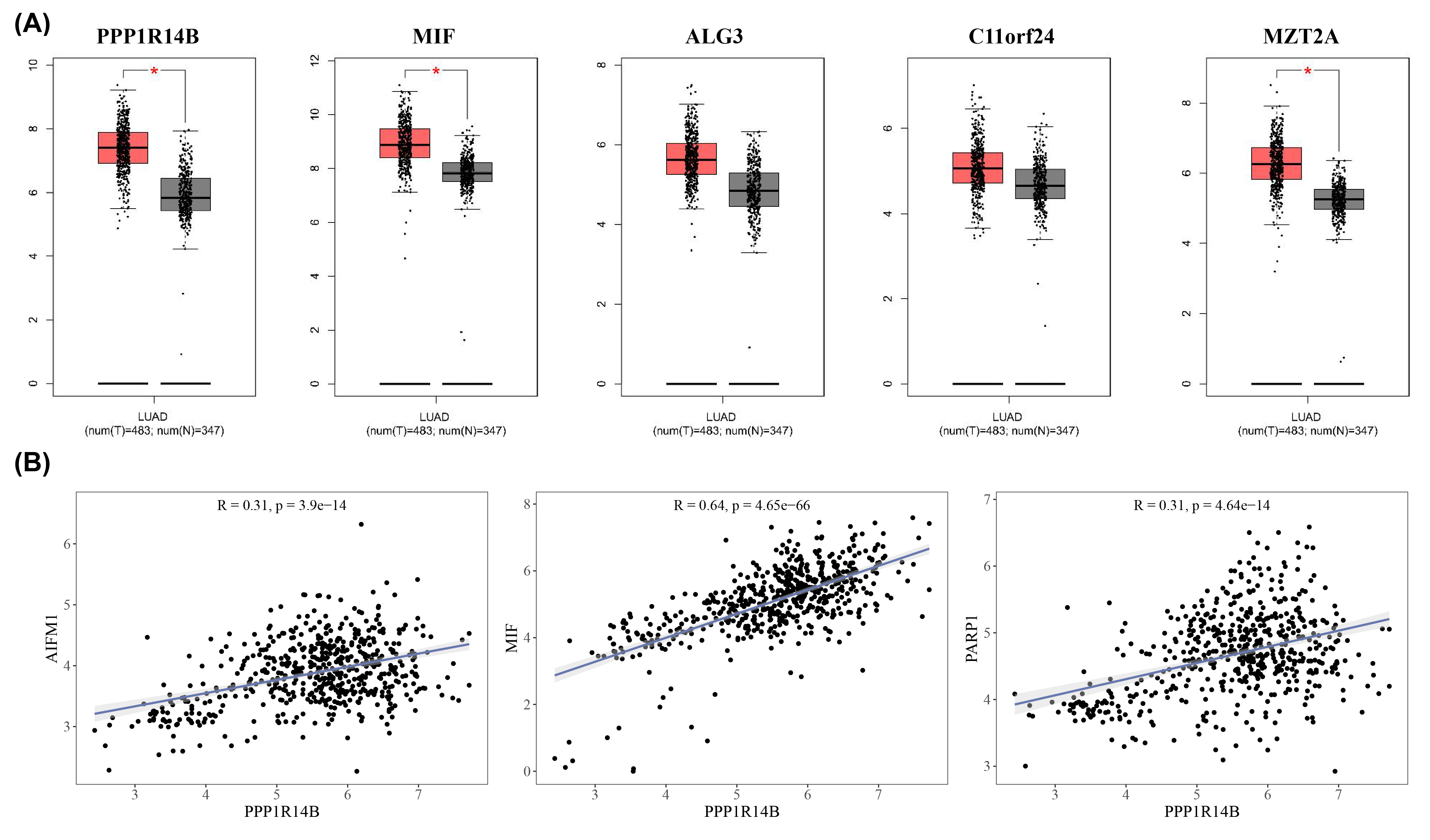

Supplement: Supplementary Figure 4 — (A) Expression of PPP1R14B, MIF, and MZT2A in the TCGA and GTEx databases. (B) Correlation analysis of PPP1R14B with core genes (PARP1, AIFM1, and MIF) in the Parthanatos pathway. [file Image4.tif]

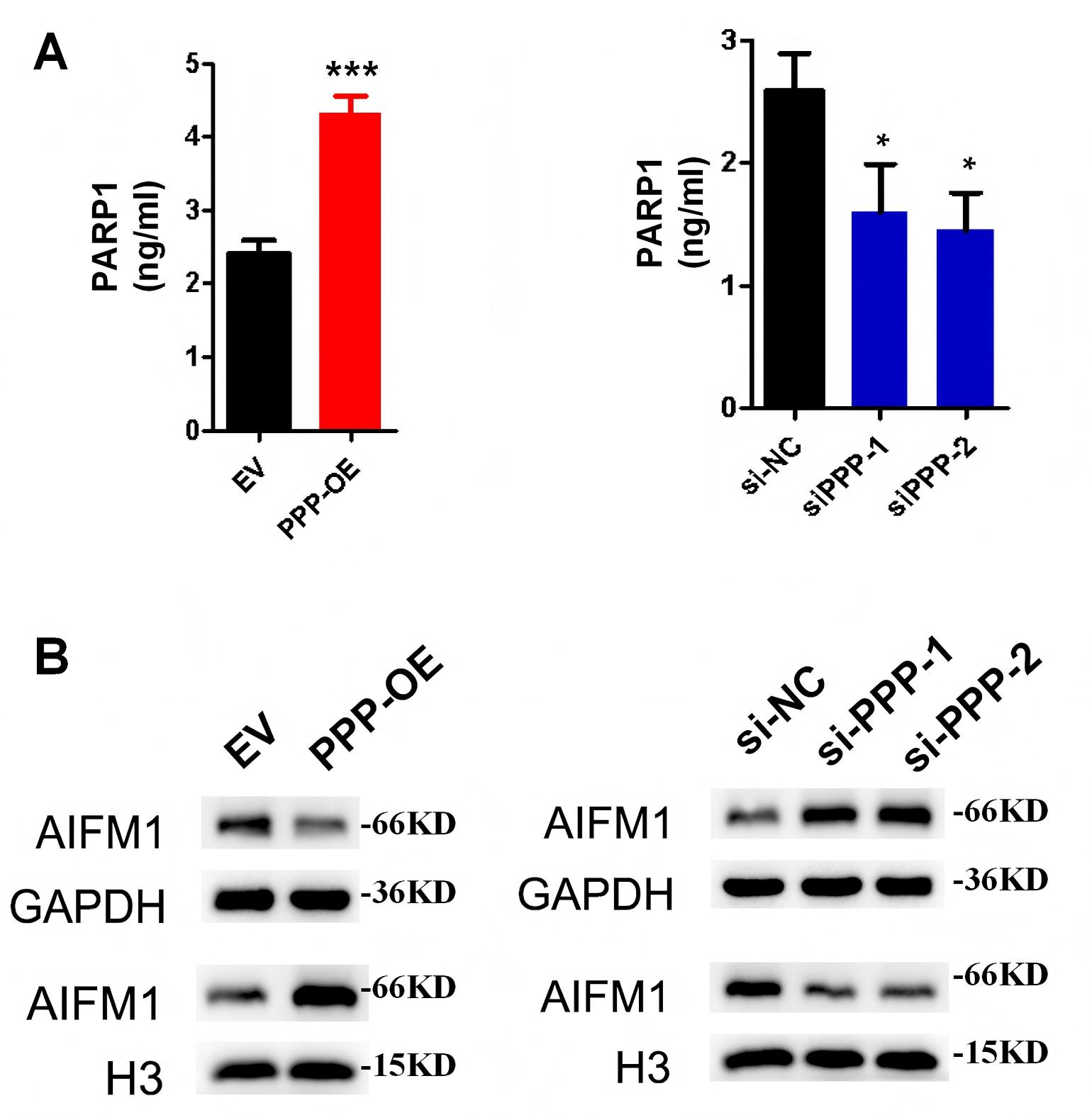

Supplement: Supplementary Figure 5 — (A) Overexpression of PPP1R14B significantly promoted PARP1 activity, downregulation of PPP1R14B resulted in a significant decrease. (B) Overexpression of PPP1R14B led to a decrease in AIFM1 expression in the cytoplasm and a concurrent increase in the nucleus(left). Knockdown of PPP1R14B increased cytoplasmic AIFM1 while decreasing its nuclear presence(right). [file Image5.jpeg]
