# Supplementary material for: Identification and validation of parthanatos-related genes in lung adenocarcinoma and construction of a prognostic risk model
Source: Front Immunol. 2026 Jul 8;17:1806560. doi: 10.3389/fimmu.2026.1806560 (PMC13388751; doi:10.3389/fimmu.2026.1806560)
Supplement: Supplementary file 6 [file Table1.docx]

Supplementary Table 1 siRNA Sequence

| siRNA Name | Sense Sequence (5'-3') | Antisense Sequence (5'-3') |
| --- | --- | --- |
| siPPP-1 | GCAUCCAGUUCUCCUAUAATT | UUAUAGGAGAACUGGAUGCTT |
| siPPP-2 | GGAAGAGCCUUGAAUACCATT | UGGUAUUCAAGGCUCUUCCTT |
