# Supplementary material for: Identification and validation of parthanatos-related genes in lung adenocarcinoma and construction of a prognostic risk model
Source: Front Immunol. 2026 Jul 8;17:1806560. doi: 10.3389/fimmu.2026.1806560 (PMC13388751; doi:10.3389/fimmu.2026.1806560)
Supplement: Supplementary file 7 [file Table2.docx]

Supplementary Table 2 RT qPCR Primer Sequence

| Target Gene | Alignment(5'-3') |
| --- | --- |
| PPP1R14B | Forward Primer:GGCAGGGAGAACTACGACAT  Reverse Primer: TCTCGTGCTCCTTGTTCTCC |
| MIF | Forward Primer: ACCAGCTCATGGCCTTCG  Reverse Primer: CTTGTAGGAGCGCGTTCATG |
| MZT2A | Forward Primer: GAAAGCAGCAGTTCGTCACC  Reverse Primer: TCCTCCTCCTCTTCCTCCTC |
| GAPDH | Forward Primer: GTCTCCTCTGACTTCAACAGCG  Reverse Primer: ACCACCCTGTTGCTGTAGCCAA |
