# Supplementary material for: Identification and validation of parthanatos-related genes in lung adenocarcinoma and construction of a prognostic risk model
Source: Front Immunol. 2026 Jul 8;17:1806560. doi: 10.3389/fimmu.2026.1806560 (PMC13388751; doi:10.3389/fimmu.2026.1806560)
Supplement: Supplementary file 9 [file Table4.docx]

**Supplementary Table 4:** The C-index and AUC values of the top 10 models

| Model | Cindex（Training） | 1year_AUC | 3year_AUC | 5year_AUC |
| --- | --- | --- | --- | --- |
| CoxBoost+RSF | 0.920221868 | 0.9553 | 0.9736 | 0.9584 |
| RSF | 0.918038591 | 0.9525 | 0.9715 | 0.9588 |
| Lasso+RSF | 0.91711414 | 0.9509 | 0.9722 | 0.9612 |
| StepCox[both]+RSF | 0.871737377 | 0.9073 | 0.9389 | 0.9468 |
| StepCox[backward]+RSF | 0.87144234 | 0.9054 | 0.9387 | 0.9459 |
| RSF+GBM | 0.704392124 | 0.73 | 0.7562 | 0.7757 |
| CoxBoost+GBM | 0.703743042 | 0.7309 | 0.7564 | 0.7725 |
| Lasso+GBM | 0.69978954 | 0.7242 | 0.7498 | 0.7662 |
| GBM | 0.699691194 | 0.7245 | 0.7534 | 0.7678 |
| StepCox[both]+GBM | 0.679028737 | 0.7018 | 0.7234 | 0.7461 |
